# Increased hydropower but with an elevated risk of reservoir operations in India under the warming climate

**DOI:** 10.1016/j.isci.2023.105986

**Published:** 2023-01-14

**Authors:** Dipesh Singh Chuphal, Vimal Mishra

**Affiliations:** 1 Civil Engineering, Indian Institute of Technology (IIT) Gandhinagar, Gandhinagar, India; 2 Earth Sciences, Indian Institute of Technology (IIT) Gandhinagar, Gandhinagar, India

**Keywords:** Earth sciences, Atmospheric science, Energy resources

## Abstract

Hydropower is a significant contributor to clean global electricity generation; therefore, it plays a crucial role in climate change mitigation. Notwithstanding major hydropower dams in India are in diverse climatic regions and exposed to risks because of the warming climate, potential changes in hydroclimate remain largely unexplored. Using observations and climate projections, we demonstrate the hydroclimatic changes in the upstream catchments and their implications for the hydropower generation of 46 major hydropower dams in India. A warmer (up to 5.0°C) and wetter projected climate with a substantial increase (5.0–33%) in precipitation will lead to an increased (7–70%) inflow to reservoirs of major dams. Increased inflow will enhance (9–36%) the hydropower production for most dams in the future, with a more prominent rise in central India dams. A simultaneous rise in extreme inflow and high reservoir storage conditions is projected under future climate for most dams. However, future climate changes project a favorable hydroclimate for hydropower production, with the associated risks related to extremes.

## Highlights

• Examined the projected hydroclimate changes in the watershed of India’s hydropower dams
• Higher dam inflow and increased hydropower are projected for hydropower dams in India
• Considerable adaptation measures will be needed to tackle the extremes in the future

## Graphical abstract

**Figure:**
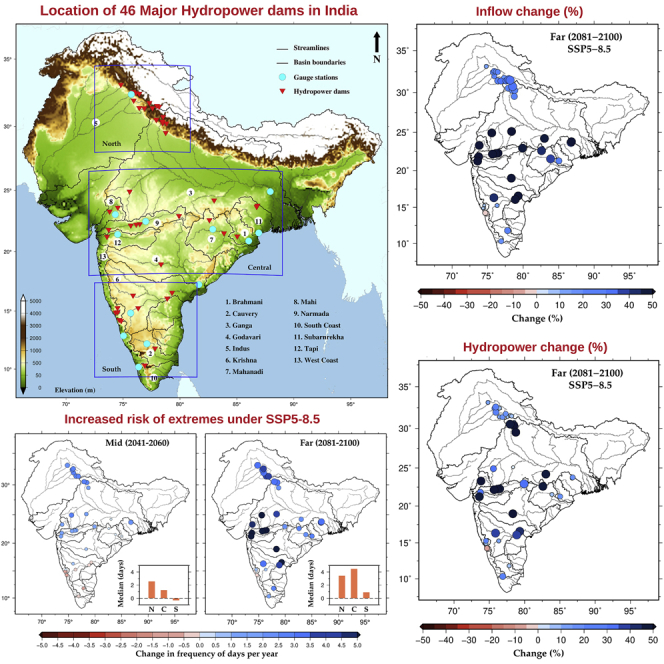

## Introduction

The energy sector will likely play a crucial role in climate mitigation and the net-zero target.^1^ India is the third largest energy-consuming nation globally, with a per capita energy consumption of 1208 kWh in 2020.^2^ About 51% of energy in India is generated from traditional energy sources,^3^ which emit greenhouse gases (GHG). Sustainable energy (wind, solar, and hydropower) can help mitigate climate change.^4^ However, hydropower, a significant source of sustainable energy, has received considerable attention for its role in climate change mitigation.^5^^,^^6^^,^^7^ Hydropower accounts for the second-largest share (13%) of the total energy produced in India,^8^ and dominates the sustainable energy share.

Climate change can significantly alter global precipitation patterns and water resource availability.^9^^,^^10^^,^^11^ Streamflow variability across the major global river basins is projected to change because of a warming climate.^12^^,^^13^ Hydropower is susceptible to a warming climate^14^^,^^15^ because of changes in reservoir storage and streamflow.^16^ Thus, climate change can significantly alter the global hydropower generation capacity.^17^^,^^18^^,^^19^ Indian sub-continental river basins (Ganga and Indus) are sensitive to a warming climate.^9^^,^^20^ However, the impacts of climate change on India’s hydropower potential remain unrecognized. Ali et al.^21^ reported a significant increase in the hydropower potential of seven hydroelectric dams in India compared to the baseline period based on the CMIP5 projections. Therefore, the impact of future climate on the currently installed hydropower systems needs to be examined to stabilize the electricity supply and proper functioning of power plants in the future.

Global hydropower potential under climate change has been widely examined.^17^^,^^18^^,^^19^ However, a regional assessment of hydropower potential in India under climate change remains limited.^21^^,^^22^ Ali et al.^21^ focused only on a small number of hydroelectric dams and did not examine the regional impacts of future climate on hydropower systems. The impacts of climate change on streamflow and hydropower potential may vary across regions in India, which remain unexplored. In addition, alternative risks to projected changes in hydropower potential have not been examined. For instance, extreme precipitation and floods have increased in the past^23^^,^^24^; however, it remains unclear if the increase in precipitation can directly translate to an increase in hydropower potential without posing challenges to reservoir operations. Using observed forcing and CMIP6 projections, we examine the changes in hydropower potential under the projected future climate and the risk of increased frequency of high flow.

## Results

### Climate projections and hydroelectric dams in India

**Figure 1:**
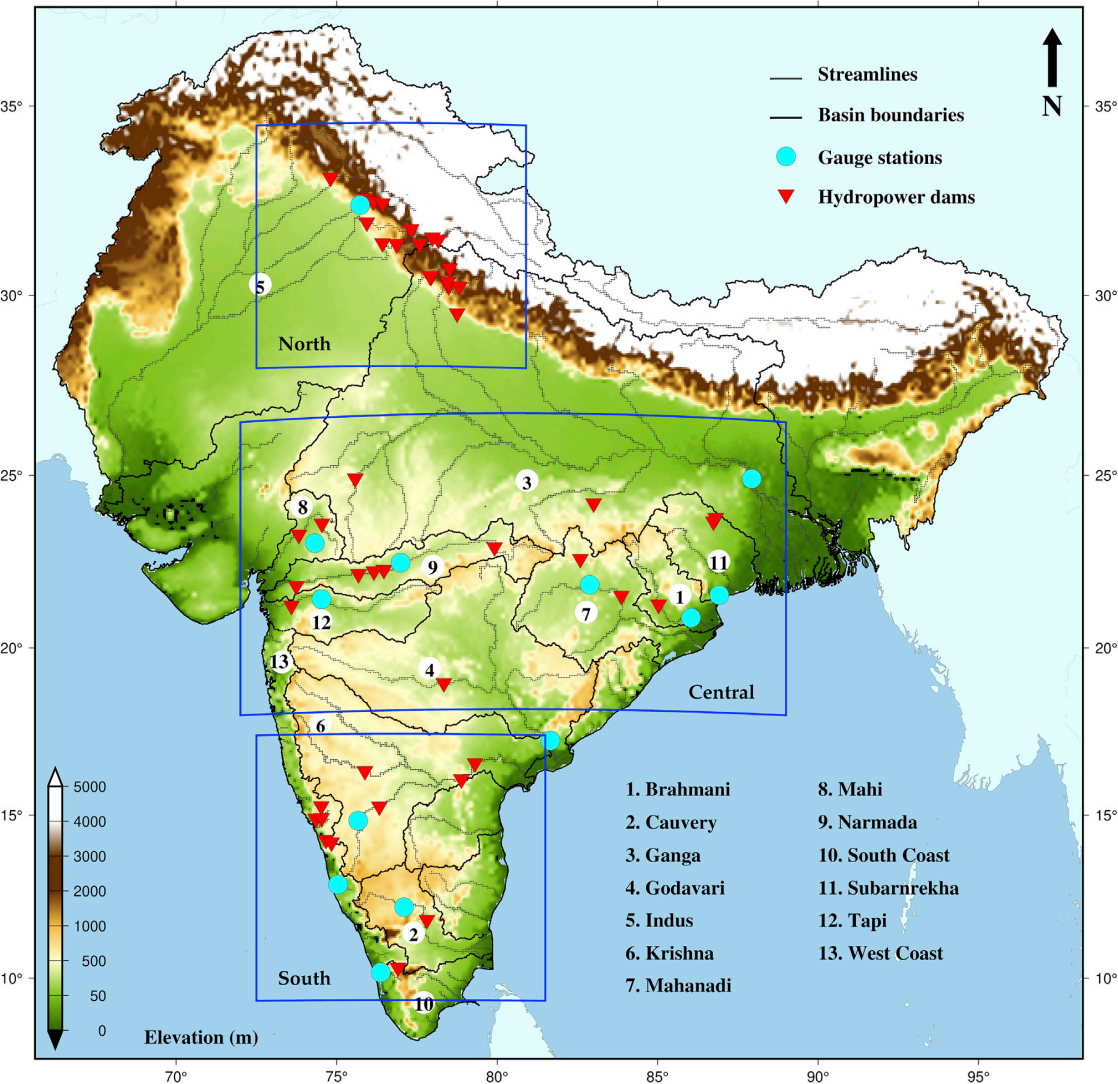
Location of hydropower dams in different river basins in India Map shows the location of selected streamflow gauging stations (cyan) and large hydropower dams (red) in 13 Indian sub-continent river basins (white circles). The black and gray lines represent basin boundaries and major streamlines in the river basins, respectively. Shaded color represents the elevation (m) from mean sea level. Blue boxes show dams in North, Central, and South India.

**Figure 2:**
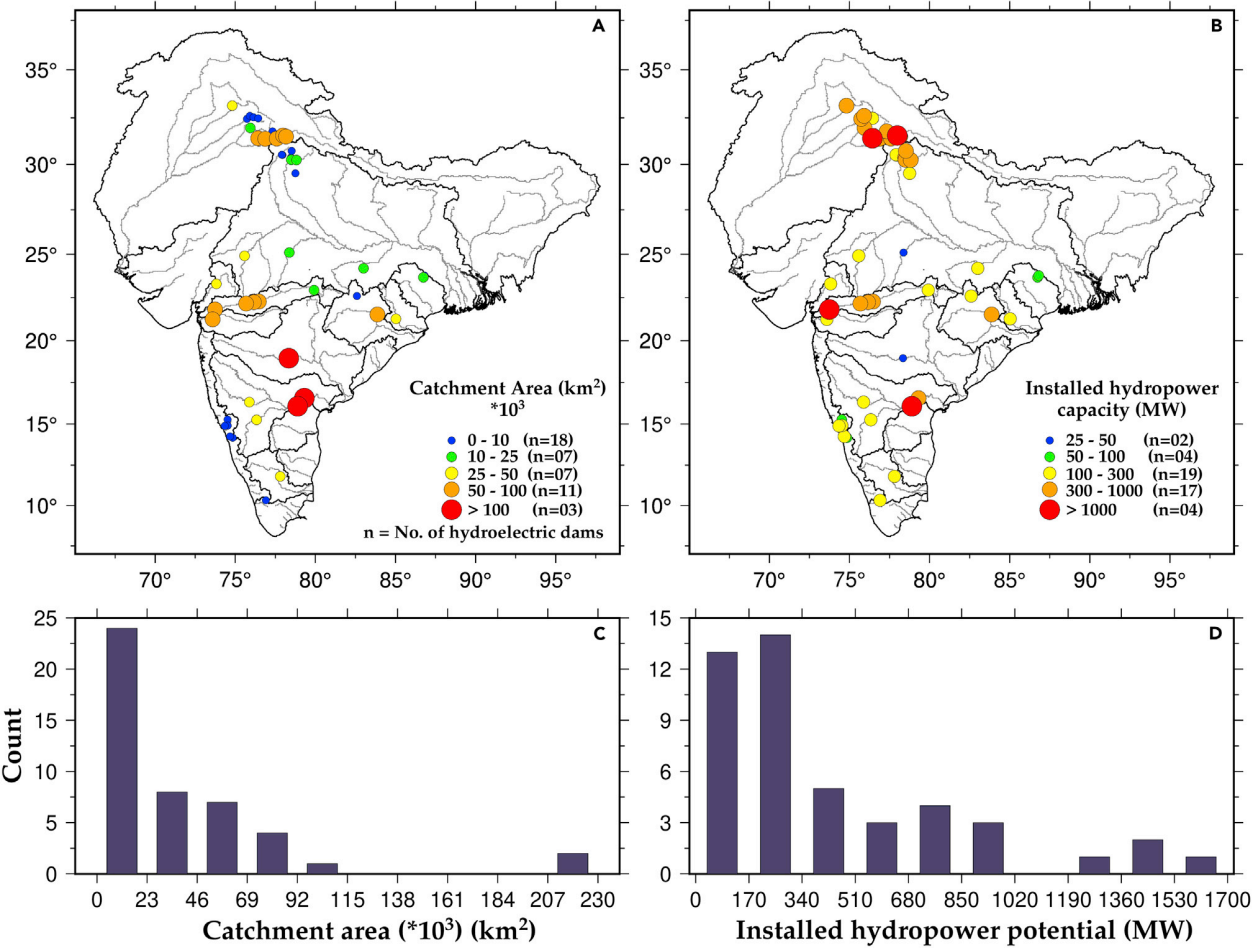
Upstream catchment area and installed hydropower potential of the major dams in India (A and B) Catchment area (km^2^) upstream of the hydropower dams and their installed hydropower capacity (MW). (C and D) Distribution of dams based on their upstream catchment area (km^2^) and installed hydropower potential (MW).

We selected 46 hydropower dams with more than 25 MW of installed hydropower located in the thirteen Indian sub-continental river basins (Figure 1). A majority of the selected dams are clustered in the north, central, and southern parts of the country. We, therefore, categorized these dams by their location in North India, Central India, and South India based on the information from the National Register of Large Dams (NRLD) [Figure 1, Table S1]. Because the selected dams are large in terms of their installed hydropower capacity and located across the country, these can provide information related to regional variability in the changes in hydropower and inflow. The catchment area upstream of the dams varies between 700 and 220,000 km^2^. At the same time, the installed hydropower capacity (IHC) of the dams ranges between 33 and 1670 MW (Figure 2). Bhakra Nangal, Naptha Jhakhari, Karcham, Sardar Sarovar, Nagarjuna Sagar, and Srisailam have higher hydropower potential (Figure S1B). Dams in Mahanadi, Narmada, and Krishna river basins receive higher inflow (Figure S1A). The mean inflow and mean hydropower potential for most dams are less than 750 m^3^/s and 550 MW, respectively (Figures S1C and S1D).

Project changes in precipitation and temperature in the upstream catchment of the major dams can influence streamflow variability,^20^^,^^25^ which can alter hydropower potential. We estimated multimodel ensemble mean changes in precipitation and temperature for upstream catchments of hydroelectric dams across India using the bias-corrected projections from CMIP6-GCMs (see STAR Methods for more details). The ensemble mean changes were estimated for the near (2021–2040), mid (2041–2060), and far (2081–2100) periods against the reference period (1995–2014) for the two scenarios (SSP1-2.6 and SSP5-8.5).

Catchments upstream of all the selected dams are projected to experience substantial warming in the future under both scenarios (Figure S2–S4). Compared to Central and South India, North India is projected to experience higher warming in the future.^26^^,^^27^^,^^28^ The highest warming (∼5°C) is projected in North India, whereas the projected warming for central and south India is around 3–4°C (Figure S4D). The projected warming in the near period ranges between 0.5 and 1.0°C and 0.50 to 1.3°C for the SSP1-2.6 and SSP5-8.5 scenarios, respectively (Figure S2, Table S2). Similarly, the mid-period warming ranges between 0.9 and 1.4°C and 1.5 to 2.5°C for the SSP1-2.6 and SSP5-8.5, respectively (Figure S3, Table S2). The highest warming is projected under the SSP5-8.5 scenario for the far period (Figure S4D), which ranges between 3.3 and 5.4°C. The three regions with the majority of hydropower dams have different warming levels projected (Figures S2–S4), which can influence the hydrology of the upstream catchments.

Similar to the substantial warming, most catchments upstream of the hydropower dams are likely to witness increased precipitation in the projected climate (Figures S5–S7). Among the three regions, North and Central India are projected to receive a higher increase in precipitation than southern India (Table S3). The projected changes in precipitation in the upstream catchment area of hydropower dams show considerable spatial variability within the central and southern Indian regions (Figures S5–S7). Ensemble mean projected changes in rainfall vary between 1.5 and 26.2% under SSP1-2.6 and between −2.5 and 20.4% under SSP5-8.5 in the near period (Table S3). A more substantial increase in precipitation is projected in the mid and far periods under both scenarios (Table S3). Most catchments upstream of the dams are projected to witness substantial warming and increased precipitation, which can alter the flow regime with implications on inflow to the dams and hydropower production.

### Projected changes in inflow to dams and hydropower

**Figure 3:**
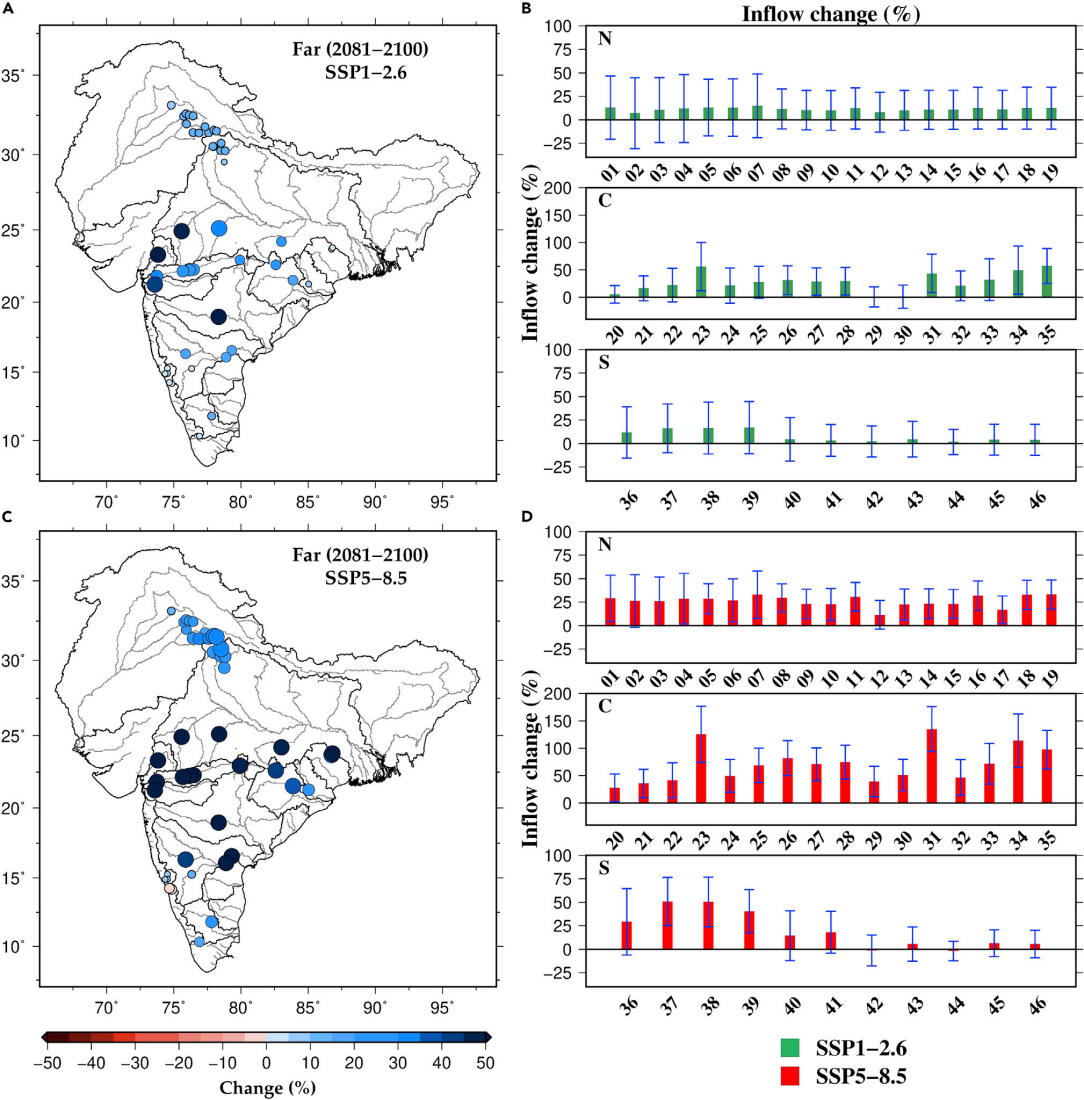
Projected change in inflow to major hydropower dams in India (A and C) Multimodel ensemble mean projected changes in inflow to the major hydropower dams in the far period (2081–2100). Changes in inflow (%) were estimated against the historical reference period (1995–2014) under SSP1-2.6 and SSP5-8.5. Hydropower dams with higher projected increase in inflow are represented with larger circles. (B and D) Projected change in inflow to hydropower dams in North (N), Central (C), and South (S) India under SSP1-2.6 and SSP5-8.5. The intermodel variation (uncertainty based on one SD) among the five CMIP6-GCMs is shown by error bars. The names of hydropower dams and other details based on the numbers in (b,d) can be seen in Table S1. For near and mid periods, see Figures S8 and S9.

Next, we estimated the projected changes in inflow simulated from the H08 and CaMa-flood models (see STAR Methods for details, Figures3, S8 and S9). Inflow for most of the dam is projected to rise in the future climate attributable to the substantial increase in precipitation (Table S4). Substantial warming and increase in precipitation are projected in the future climate; however, precipitation is the dominating factor controlling streamflow variability in the Indian sub-continental river basins.^20^^,^^25^^,^^29^ Hydropower dams in central India will receive higher inflow in the future than in northern and southern India (Figure 3). However, inflow for a few hydropower dams in Ganga, Mahanadi, Brahmani, and West-coast River basins is projected to decline in the future (Figures 3, S8, and S9, and Table S4), which can be because of rise in atmospheric water demands in response to the considerable warming^16^ compared to increase in precipitation. Ensemble mean projected changes in inflow range between −9.5 and 55.7% in the near period, −7.7 and 64.5% in the mid-period, and −1.8 and 135.0% in the far period (Table S4). Among the 46 hydroelectric dams, Ukai and Kadana dams are projected to experience a considerable increase in streamflow (more than 100%) in the future climate primarily because of rise in precipitation (Figure S7).

**Figure 4:**
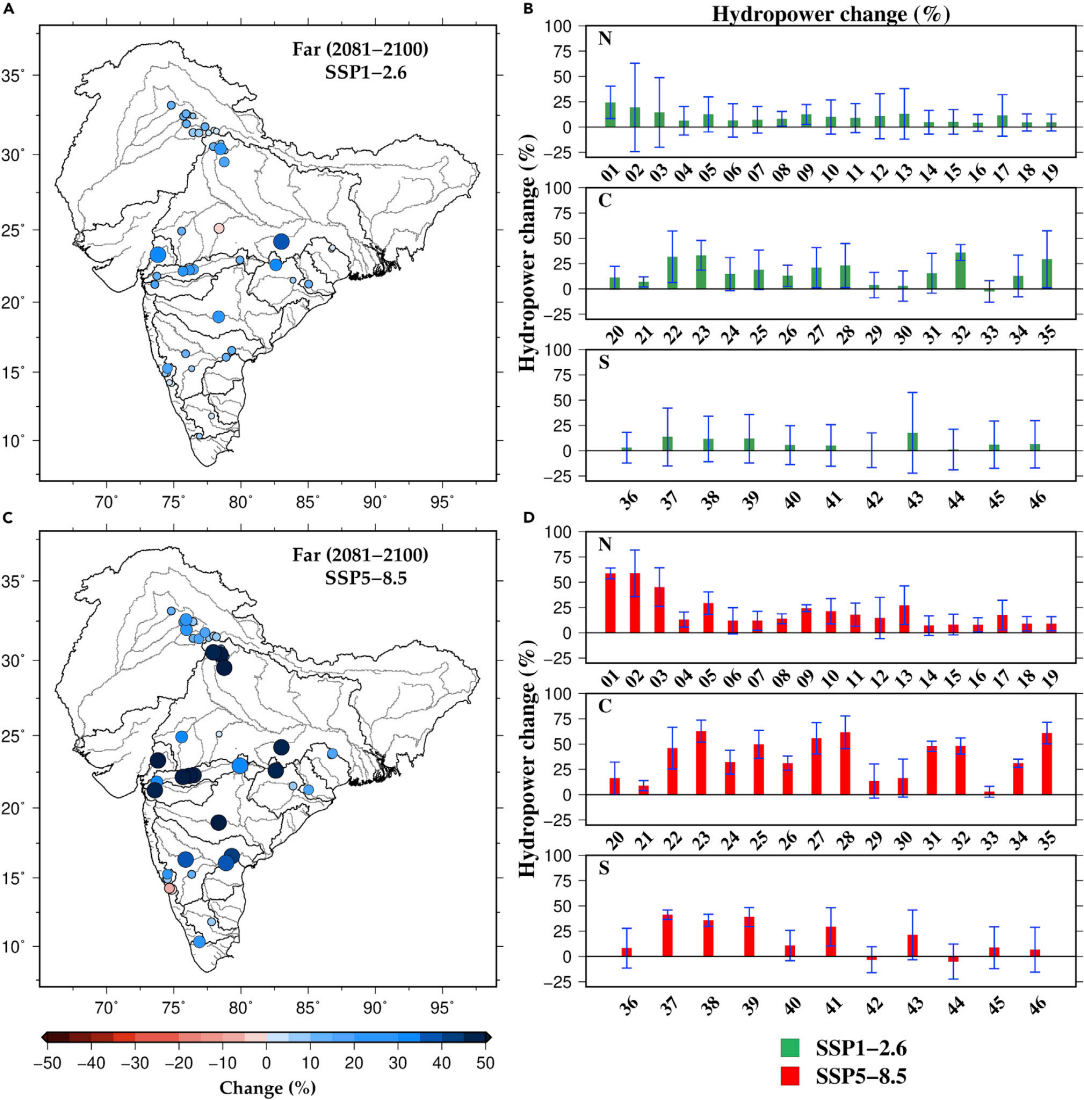
Projected change in hydropower potential of the major dams in India (A and C) Multimodel ensemble mean projected changes in hydropower of the major dams in the far period (2081–2100). Changes in hydropower (%) were estimated against the historical reference period (1995–2014) under SSP1-2.6 and SSP5-8.5. Hydropower dams with higher projected increase in hydropower are represented with larger circles. (B and D) Projected change in hydropower for dams in North (N), Central (C), and South (S) India under SSP1-2.6 and SSP5-8.5. The intermodel variation (uncertainty based on one SD) among the five CMIP6-GCMs is shown by error bars. The names of hydropower dams and other details based on the numbers in (b,d) can be seen in Table S1. For near and mid periods, see Figures S10 and S11.

The projected rise in precipitation and temperature results in enhanced inflow to reservoirs of the major hydropower dams in India which can influence hydropower production. We estimated the projected changes in DHP for all 46 dams using the multimodel ensemble mean streamflow for the near, mid, and far periods (Figure 4, Tables S4 and S5). DHP projections are consistent with inflow projections for most dams, indicating a projected rise in hydropower potential in the future (Figures 4, S10, and S11). However, a few dams show a substantial intermodel uncertainty in inflow and DHP under the projected future climate (Tables S4 and S5). The projected change in DHP is the highest in the far period and the lowest for the near period (Figures 4D and S10D). Projected change in DHP ranges between −6.2 and 39% in the near period,−24 and 58.2% in the mid-period, and −5.0 and 62.8% in the far period (Table S5). DHP is projected to rise by more than 50% in Tehri, Ramganga, Kadana, Omkareshwar, Maheshwar, and Sriramsagar dams in the far period (Figure 4D). On the other hand, eight out of eleven dams in south India are projected to experience a decline in hydropower potential under SSP5-8.5 (Figures S10 and S11). Overall, dams in central India are projected to experience a more substantial increase in DHP than in the other two (north and central) regions (Figure 4).

Next, we estimated multimodel ensemble mean changes in inflow and DHP for different global warming levels (1.5°C, 2°C, 3°C) [Figure S12 and Table S6)]. We estimated decades for each CMIP6-GCM under the two scenarios with corresponding global warming levels.^30^^,^^31^ The median changes in inflow to the dams in North, Central, and South India vary between 10 and 20%, 14 and 55%, and −5 and 0.5% under selected global warming levels. Changes in DHP at different global warming levels are consistent with the changes in inflow (Figure S12). For instance, the median change in DHP for the dams in North, Central, and South India ranges between 10 and 20%, 20 and 40%, and −10 and 3% under the selected global warming levels. Consistent with the inflow, dams in south India are likely to witness a reduction in DHP at 1.5°C and 3°C global warming levels (Figure S12). The mean increase in DHP is the highest at a 3°C global warming level in all three regions. Based on the CMIP6 projections, we find a favorable condition for hydropower production for most of the dams in India under the warming climate, which can be attributed to a considerable rise in inflow to reservoirs. The increase in inflow is mainly because of a substantial increase in precipitation in the upstream catchments. Under the warming climate, atmospheric water demand is also projected to rise.^32^ However, the projected increase in rainfall can offset the impacts of warming. Notwithstanding the favorable changes in inflow and hydropower in the future, it remains unclear whether these changes can also pose challenges in reservoir operations.

### Projected increase in high flow and risk to reservoir operations

We computed the projected changes in high inflow events under the warming climate for all the dams to examine the potential risks to reservoir operation (Figure S13). The high-flow events were selected considering the 95^th^ percentile threshold for the historical period (1995–2014). The frequency of high-inflow events is projected to rise substantially for most hydropower dams in the future (Figure S13). The highest increase in high inflow events to hydropower dams is projected in the far period under the highest emission scenario of SSP5-8.5 (Figure S13). However, the frequency of high inflow events is projected to decline for the dams in south India in the near and mid periods (Figure S13). The high-inflow events can pose challenges for the reservoir operations depending on the reservoir storage and magnitude of the events. The increased water availability during these high-flow events when the reservoir is full can pose challenges for controlling flooding in downstream regions. Most reservoirs receive significant storage during the summer monsoon season (June to September) after a considerable decline in storage in the pre-monsoon season to meet the irrigation demands.^33^ Most of the dams in India are multi-purpose and can be used for hydropower, flood control, and irrigation water supply. The challenges for reservoir operations primarily arise during the peak monsoon season (July-August), when reservoirs are full and cannot accommodate the high inflow.^34^

**Figure 5:**
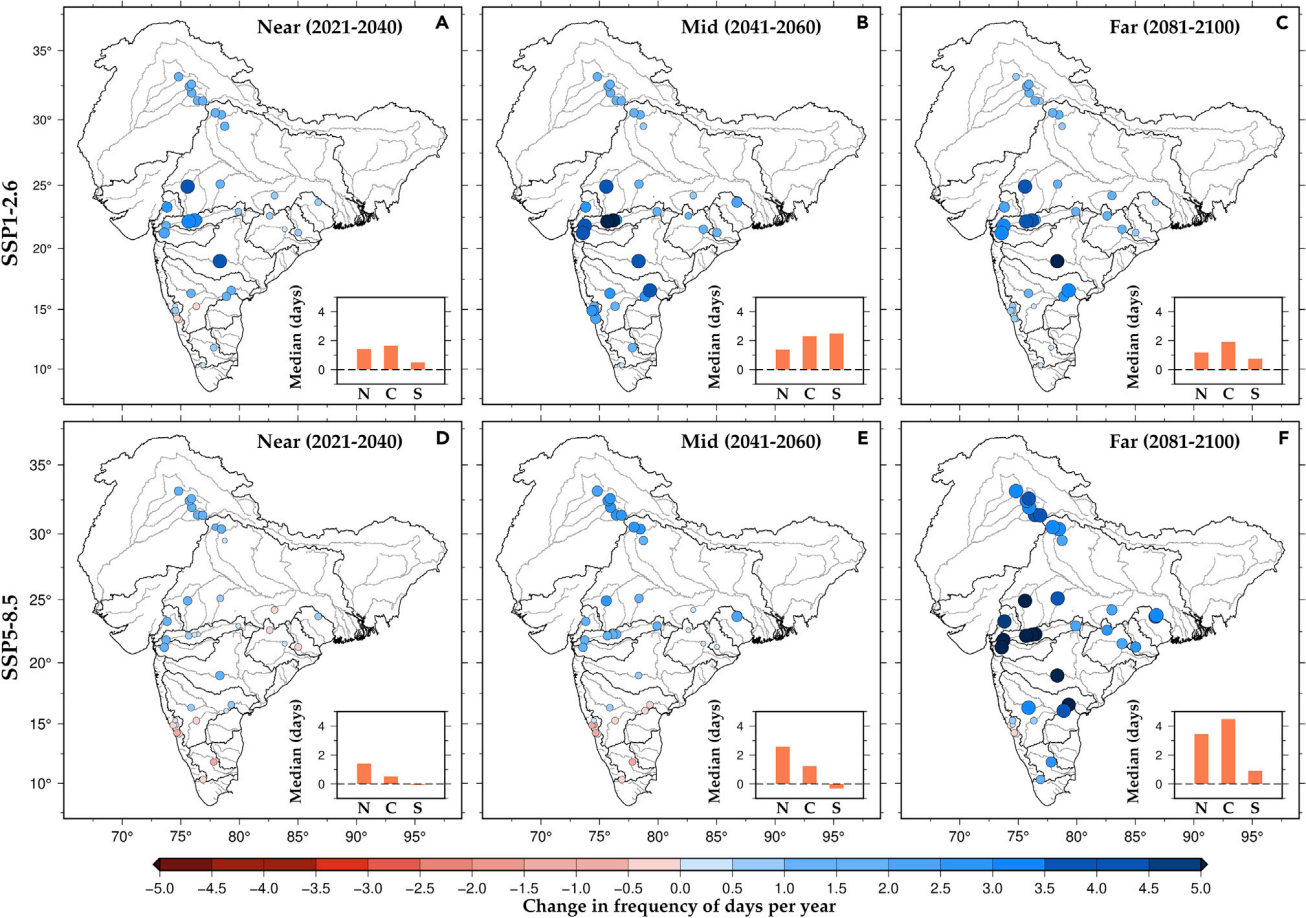
Projected change in the frequency of high inflow and high reservoir storage in the major hydropower dams in India Multimodel ensemble mean changes in the frequency of days (reservoir storage >95^th^ percentile & inflow >95^th^ percentile) per year were estimated for the near, mid, and far periods against the historical reference period. Median changes (days) in each region (North, Central, and South) are shown as bars (orange) in the panel inside the figures for both SSPs. For projected change in the frequency of high inflow, see Figure S13.

Next, we estimated the frequency of days when both reservoir storage and dam inflow exceed the 95^th^ percentile to examine the potential risk to reservoir operations. High inflow when reservoir storage is about full can pose challenges for reservoir operations. In the absence of reliable early warning and forecast systems, high inflow when reservoirs have already reached their total capacity may require quick release, which can cause flooding in the downstream regions.^35^ In addition, the oversupply to reservoirs can also lead to risks of dam breaks.^36^^,^^37^ Therefore, the rise in the high inflow events (Figure S13) because of the heavy precipitation in the upstream catchment of the hydropower dam with the higher reservoir storage imposes risks to reservoir operations, causing the sudden opening of the dam gates and flooding in the downstream low-lying areas.^38^ Indian sub-continental river basins during the observed period witnessed such extreme conditions in the past. For instance, the Machchhu dam failure in 1979 and the Kerala floods in 2018 were associated with reservoir storage.^39^^,^^40^ Simultaneous occurrence of high inflow when reservoirs have high storage more often because of the warming climate (Figure 5). Reservoir operations can be challenging because of increased high inflow events, especially for hydropower dams in central India. On the other hand, hydropower dams in south India are projected to experience high reservoir storage and high inflow condition less frequently during the mid and far periods (Figures 5D and 5E).

## Discussion

We estimated the changes in the hydropower potential across different regions of India under a warming climate. The climate change impacts on hydropower potential differ among regions, which project an increase in hydropower potential in North and Central India and a decline in hydropower potential for a few hydropower dams in South India. Hydropower production predominantly depends on precipitation and streamflow.^14^ The increase in hydropower potential can be attributed to increased inflow in the future climate. Streamflow in North India river basins is greatly influenced by snow and glacial meltwater. Projected substantial warming in North India is likely to reduce snow and glacial storage, reducing snowmelt water contribution to the streamflow in the long run.^26^^,^^28^^,^^41^ However, an increase in future inflow to reservoirs in North India is because of substantial increase in rainfall in the future climate. Furthermore, the projected decline in hydropower with increased precipitation for a few dams can be attributed to the higher evapotranspiration rate than precipitation in the future.^16^

A more robust increase in inflow to dams is projected based on CMIP6-GCMs compared to the previous studies.^21^^,^^22^ The projected increase in hydropower potential (up to 60%) is considerably higher than the previously reported [up to 25%].^21^ The increase in high-flow events because of rise in extreme precipitation is projected for India under future climate, which is consistent with the previous findings.^42^ The projected increase in extreme inflow events will impose difficulties in the management of reservoirs for flood control, hydropower production, and water supply.^43^ In addition, the regional impacts of climate change on hydropower generation in India were not discussed in the previous studies.^21^ Our results show considerable regional variability in the impact of climate change on hydropower production under the warming climate across India.

We used hydrological model simulations for the observed and projected future climate for runoff and streamflow. We find a considerable intermodal variation in the inflow and hydropower projections highlighting the uncertainty because of climate models (please see supplemental tables for further details). The ability of existing GCMs is limited in simulating the key features of the monsoonal climate because of coarser spatial resolution. Regional climate models at higher resolution that can better resolve topography can strengthen the projections.^44^ The uncertainty associated with the hydrological models can mainly be because of input datasets and model parametrization.^45^^,^^46^ The ensemble projections from multiple hydrological models can reduce parametric uncertainty in the hydroclimatic projections.^29^^,^^47^ Other factors like land use/land cover change and construction of new reservoirs upstream of the existing hydropower dams can also impact river flow regimes and sediment load to the reservoirs,^48^^,^^49^^,^^50^^,^^51^ which can influence hydropower potential in the future. The projected increase in high-flow events can increase sedimentation in the reservoir, which can further reduce the storage capacity.^52^ We used a simplified reservoir operating algorithm for computing release that does not account for future irrigation and other demands, which can also affect hydropower production.^53^ Despite these uncertainties and limitations, our findings provide crucial information regarding the future changes in hydropower potential and water resources in the Indian sub-continental river basins.

Projected warmer and wetter conditions will increase most dams’ hydropower potential. However, the impact of future climate on hydropower is not uniform across the dams. For instance, our results indicate that a decline in hydropower potential for a few hydropower dams in south India is projected. Dams in Tapi, Narmada, Mahi, Godavari, and Mahanadi river basins show a more remarkable rise in future hydropower potential. Based on selected hydroelectric dams, India is projected to increase hydropower potential (by 10-23%). However, the frequency of high inflow events is projected to rise for most hydropower dams, with the frequency of high reservoir storage. Reservoirs with high storage are projected to witness extreme inflow conditions more often in the future, leading to challenges for reservoir operations. Our findings can provide crucial insights related to projected changes in hydroclimate and hydropower for the major dams in India to planners and policymakers. In addition, we highlight the challenges and opportunities associated with climate change mitigation and adaptation in the context of hydropower in India under the warming climate. Our findings emphasize the need of reliable early warning systems that can assist reservoir operations in the future.

## STAR★Methods

### Key resources table

**Table:** 

REAGENT or RESOURCE	SOURCE	IDENTIFIER
**Deposited data**		

Observed Meteorological data	IMD, Sheffield et al.^54^	https://www.imdpune.gov.in; https://doi.org/10.1175/JCLI3790.1
Observed streamflow and storage data	India WRIS	https://indiawris.gov.in/
Details of hydroelectric dams	NRLD	http://cwc.gov.in/sites/default/files/nrld-2019.pdf
Climate model data	ESGF	https://esgf-node.llnl.gov/search/cmip6/

**Software and algorithms**		

Source codes	Authors	https://github.com/DIPESHSINGHCHUPHAL/Hydropower-India.git

### Resource availability

#### Lead contact

Further information and requests for resources should be directed to and will be fulfilled by the lead contact, V. Mishra (vmishra@iitgn.ac.in).

#### Materials availability

This study did not generate new unique reagents.

### Method details

#### Data

We obtained daily precipitation and minimum and maximum temperature for the observed period. We used daily long-term gridded observed meteorological data provided by India Meteorological Department (IMD) at 0.25° spatial resolution for the Indian region. The precipitation dataset was developed by taking observations from more than 6900 rain gauges in India.^55^ Srivastava et al.^54^ developed gridded daily temperatures (maximum and minimum) at 1° spatial resolution using observations from 395 gauge stations in India. Daily maximum and minimum temperatures were re-gridded at 0.25° using bilinear interpolation to make them consistent with precipitation. We used daily long-term observed meteorological data from the gridded database developed by Sheffield et al.,^54^ which is available at 0.25⁰ spatial resolution for the regions outside India which are not covered by the observational network of IMD. Gridded precipitation and temperature datasets from Sheffield et al.^54^ compare well with the gridded observations from IMD for the Indian region.^56^

We selected the five global climate models (BCC-CSM2-MR, INM-CM5-0, MIROC6, NorESM2-MM, TaiESM1) that were part of the Coupled Model Intercomparison Project (CMIP)-6 [CMIP6-GCMs] to examine the changes in streamflow and hydropower potential under the projected future climate (Table S7). The selected GCMs capture the observed seasonal cycle of temperature and precipitation over the Indian monsoon region (Figure S14). However, INM-CM5-0 underestimated the monthly temperature over the Indian region (Figure S14, Table S8). We considered the two shared socioeconomic pathways (SSP1-2.6 and SSP5-8.5). SSP1-2.6 is a low-emission scenario, while SSP5-8.5 is the scenario that considers high radiative forcing (8.5 W/m^2^) by the end of the 21^st^ century. We used SSP1-2.6 and SSP5-8.5 to consider the most optimistic and worst-case scenarios. The difference between these two scenarios can provide us with information regarding the benefits of climate change mitigation. We bias-corrected the CMIP6 projections for the five GCMs to remove the bias using the method developed by Hempel et al.,^57^ which has been widely used in ISIMIP assessments.^21^^,^^58^^,^^59^ The proposed bias correction method preserves the long-term climate signal in the GCM data. The bias correction was performed using observations from IMD for the Indian region and Sheffield et al.^54^ for the region outside India. We estimated the percentage bias (p-bias) and normalized root-mean-square error (NRMSE) between CMIP6 precipitation and observed precipitation data to examine the accuracy of the bias-correction method (Figures S15–S17, Table S8).

We considered reservoir storage and run-of-river hydroelectric plants in our analysis. We did not consider pumped storage as it is not a major contributor to hydropower production in India. We selected 46 large hydroelectric dams that have more than 25 MW of installed power across India from 13 river basins in the Indian sub-continent (Figure 1). Out of 46 hydroelectric dams, 36 are reservoir storage types, while the other 10 are functional based on run-of-river types (Table S1). Hydroelectric dams with reservoir storage were primarily selected based on the observed reservoir storage, the location of the turbine at the river’s bed, and installed hydropower capacity. Similarly, hydroelectric dams that use run-of-river were selected based on available net elevation head and installed hydropower capacity (Installed hydropower capacity, IHC >100 MW). The installed hydropower capacity of these hydroelectric dams was taken from the India Water Resources Information System (India-WRIS). We did not include the projects in the other five river basins (East coast, Northeast coast, Pennar, Sabarmati, Brahmaputra) due to the unavailability of observed data and an undeveloped hydropower system. Most run-of-river hydropower plants are in north India due to the year-round streamflow availability in the Ganga and Indus river basins.

We categorized the selected hydroelectric dams by their locations in North India, Central India, and South India based on the information in the National Register of Large Dams (NRLD) [Figure 1]. We collected the observed reservoir storage and streamflow from India-WRIS. In contrast, the monthly streamflow for Ganga and Indus river basins (transboundary) was obtained from the Center for Sustainability and the Global Environment (SAGE) as the observed streamflow data for transboundary rivers is not available publicly.

#### Hydrological modeling for streamflow simulations

We used two hydrological models (H08 and CaMa-Flood). The H08 hydrological model is a large-scale grid-based model consisting of six modules: land surface hydrology, global river routing, reservoir operations, anthropogenic water withdrawing, crop growth, and environmental flow. Hanasaki et al.^60^ recently implemented six additional schemes in the coupled H08 model. We used the land surface module, which solves the water and energy balance within each grid to generate the runoff. The land surface module uses the "bucket model" and "leaky bucket" concepts to estimate surface and subsurface runoff, while the bulk-transfer method estimates evapotranspiration.^61^ Soil moisture and snowmelt are calculated based on water and energy balance concepts, respectively.^62^ The H08 model uses soil parameter data from Harmonized World Soil Database (HWSD). We forced the H08 model with the input meteorological forcing for the observed and projected future climate at 0.25⁰ spatial and daily temporal resolutions.

The CaMa-Flood model is a large-scale distributed routing model developed by Yamazaki et al.^63^ The global river network in the CaMa-Flood model is divided into hydrological units called unit catchments to assess efficient flow. The topographic parameters are calculated using the Flexible Locations of Waterways (FLOW) method.^63^ The current version of CaMa-Flood (version 4.01) also incorporates reservoir operations. Zhao et al.^64^ reported that CaMa-Flood outperforms other routing models for reproducing historical streamflow.

We combined the H08 land surface module and the CaMa-Flood reservoir module. The H08-land model simulates the water cycle and generates the gridded runoff at the daily timescale. CaMa-Flood routes daily gridded runoff along the river network to produce gridded discharge. CaMa-Flood routing was performed considering the influence of reservoirs. The disturbed (with reservoirs) streamflow includes the influence of upstream reservoirs. The daily natural runoff generated by the H08 model was forced into the CaMa-Flood to produce the streamflow in the presence of reservoirs. We manually calibrated the models simultaneously so that the simulated streamflow compares well with the observed streamflow. We adjusted four parameters to calibrate the H08 model for each river basin: single-layer soil depth (meter), gamma (dimensionless), bulk transfer coefficient (a factor used in the calculation of potential evaporation), and tau (days). We estimated Nash-Sutcliffe Efficiency (NSE)^65^ and coefficient of determination (R2) for daily streamflow to examine the model’s performance.

We calibrated and validated the combined modeling system (H08 and CaMa-Flood) against the observed daily streamflow for 11 river basins. Due to the unavailability of daily observed streamflow for the Ganga and Indus river basins, the models were calibrated and validated against monthly streamflow. We used about 50% of the available flow observations for calibration and the remaining for the model evaluation. We evaluated the model considering the influence of reservoirs for sufficient long period to capture the role of climate variability. All the selected gauging stations from 13 river basins show NSE and R2 coefficients greater than 0.50 for the calibration and evaluation periods (Table S9, Figures S18 and S19). In addition, NSE for ten river basins was more than 0.60 during the calibration and evaluation (Table S9). The model performed comparatively well (NSE >0.70) for Godavari and Ganga river basins and satisfactory (NSE ∼0.60) for the Cauvery river basin. The model underestimates the observed streamflow during the wet periods for the Cauvery river basin. We also compared the reservoir storage simulated by CaMa-flood against the observed storage from India-WRIS to evaluate the performance of the CaMa-Flood reservoir mechanism. The CaMa-Flood simulated reservoir storage shows satisfactory NSE and R2 above 0.50 for most reservoirs (Figure S20).

#### Estimation of historical and projected hydropower potential

We estimated the monthly developed hydropower potential (DHP) of dams for historical (1995–2014) and future periods [near (2021–2040), mid (2041–2060), and far (2081–2100)]. We also estimated the decadal average DHP change for dams corresponding to 1.5, 2, and 3 °C global warming levels. We selected the decades (Table S6) for each GCM that were 1 (0.8–1.2), 2 (1.8–2.2), and 3 (2.8–3.2) °C warmer than the corresponding GCM baseline period (pre-industrial era: 1851–1900) for both SSPs.^30^^,^^31^ DHP is the maximum hydropower produced by a dam, utilizing the currently installed hydropower services like installed hydropower capacity (IHC), reservoir storage, and dam height. DHP may not reflect the actual hydropower produced because it is greatly influenced by the demands and economic elements of the current period.^66^ Due to the complexities in hydrological modeling focusing on conventional reservoir operating rules, simplified reservoir operating algorithms are generally adopted.^14^^,^^67^^,^^68^ We used the reservoir operating algorithm developed by Hanasaki et al.^67^

Hydropower produced by a dam depends on the monthly release from the dam. The variation in the monthly and yearly inflow to the dam affects the release. If the annual inflow exceeds the total reservoir storage, the monthly release fluctuates throughout the year to avoid overflowing and reservoir storage drop. Similarly, if the yearly inflow is less than the reservoir storage, the monthly release remains consistent throughout the year.^67^ The monthly release R_m,y_ (m^3^/s) based on the reservoir operating algorithm developed by Hanasaki et al.,^67^ not including irrigation, domestic, and industrial water demands, is expressed as:

$$R_{m,y}=\left\{ \begin{array}{c} k_{y}i_{y},c\geq 0.5\\ {\left(\frac{c}{0.5}\right)}^{2}k_{y}i_{y}+\left[1-{\left(\frac{c}{0.5}\right)}^{2}\right]i_{m,y},0<c<0.5\\ i_{m,y},c=0 \end{array}\right.$$

m and y represent the month and year, i_y_ represents mean yearly inflow (m^3^/s), i_m,y_ represents mean monthly inflow (m^3^/s), c = C/I_y,_ k_y_ = S_start_/αC. I_y_ represents total yearly inflow (m^3^/year), C and S_start_ represent total reservoir storage capacity (m^3^), and storage at the start of each year (m^3^), respectively. α is taken 0.85, based on the sensitivity test.^67^ Reservoir operating algorithm was framed using the concept of the operational year. The operational year begins in the post-monsoon season (October onwards) when the reservoir starts releasing water. We forced three conditions while calculating the release: i) a reservoir will only release water when the storage is more than 10% of the total reservoir storage capacity,^68^ ii) a minimum of 10% of the monthly inflow should be released from the reservoir every month, and iii) excess water from the reservoir is allowed to release during the overfilling. The DHP (MW) of the dams was then calculated as:

$$DHP\;\left(MW\right)=\left(\gamma \times R_{m,y}\times h\right)$$

DHP at any time is taken as the minimum of (γ × R_m,y_ × h) and installed hydropower capacity. γ represents the specific weight of water (kN/m^3^), and h represents the hydraulic head (m). The hydraulic head was taken as the net elevation head between the powerhouse and the dam for run-of-river dams. The hydraulic head was calculated as H=S_m_/A for reservoir storage dams, as mentioned in Ali et al.^21^ Here, S_m_ represents the mean monthly reservoir storage; A represents the reservoir area, A = C/H, and H represents the dam height, which is also the maximum of the hydraulic head (h).

## Data Availability

• Data: All the data used in this study can be obtained from the lead contact upon request. • Code: This paper does not report any original code. However, the codes used for analysis are written in MATLAB, and the links are listed in the key resources table. • Additional information: Any additional information required to reanalyse the data reported in this paper is available from the lead contact upon request.
