# Supplementary material for: Increased hydropower but with an elevated risk of reservoir operations in India under the warming climate
Source: iScience. 2023 Jan 14;26(2):105986. doi: 10.1016/j.isci.2023.105986 (PMC9900399; doi:10.1016/j.isci.2023.105986)
Supplement: Document S1. Figures S1–S20 and Tables S1–S9 [file mmc1.pdf]

iScience, Volume *26*

Supplemental information

Increased hydropower but with an elevated risk of reservoir operations in India under the warming climate

Dipesh Singh Chuphal and Vimal Mishra

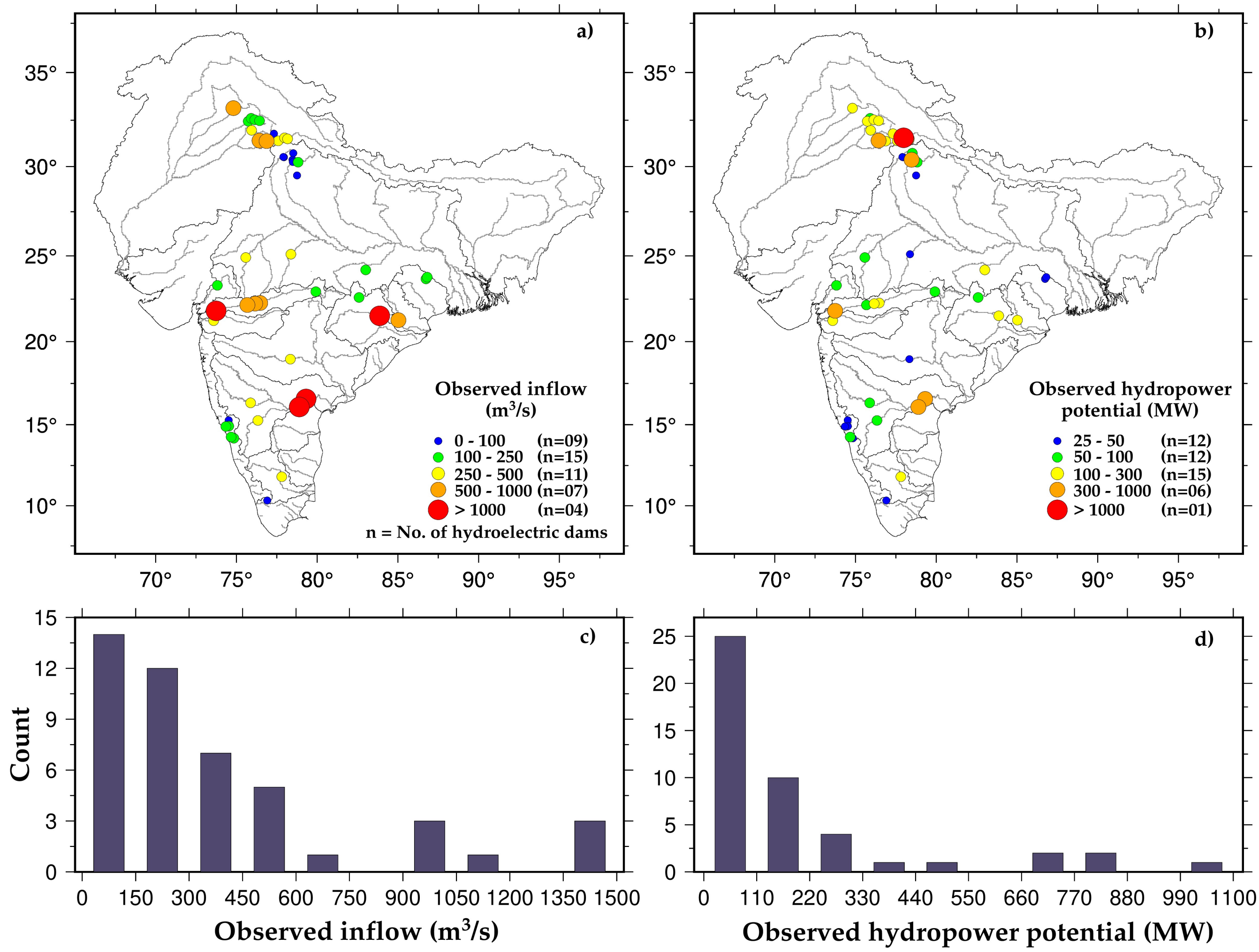

Figure S1. CaMa-Flood simulated mean inflow and mean hydropower potential of the selected hydropower dams in India. (a-b) Observed mean inflow (m^3/s) of the hydropower dams and their observed mean hydropower potential (MW) based on the meteorological data from IMD (1995-2014). (c-d) Distribution of dams based on their observed mean inflow (m^3/s) and observed mean hydropower potential (MW), Related to STAR Methods

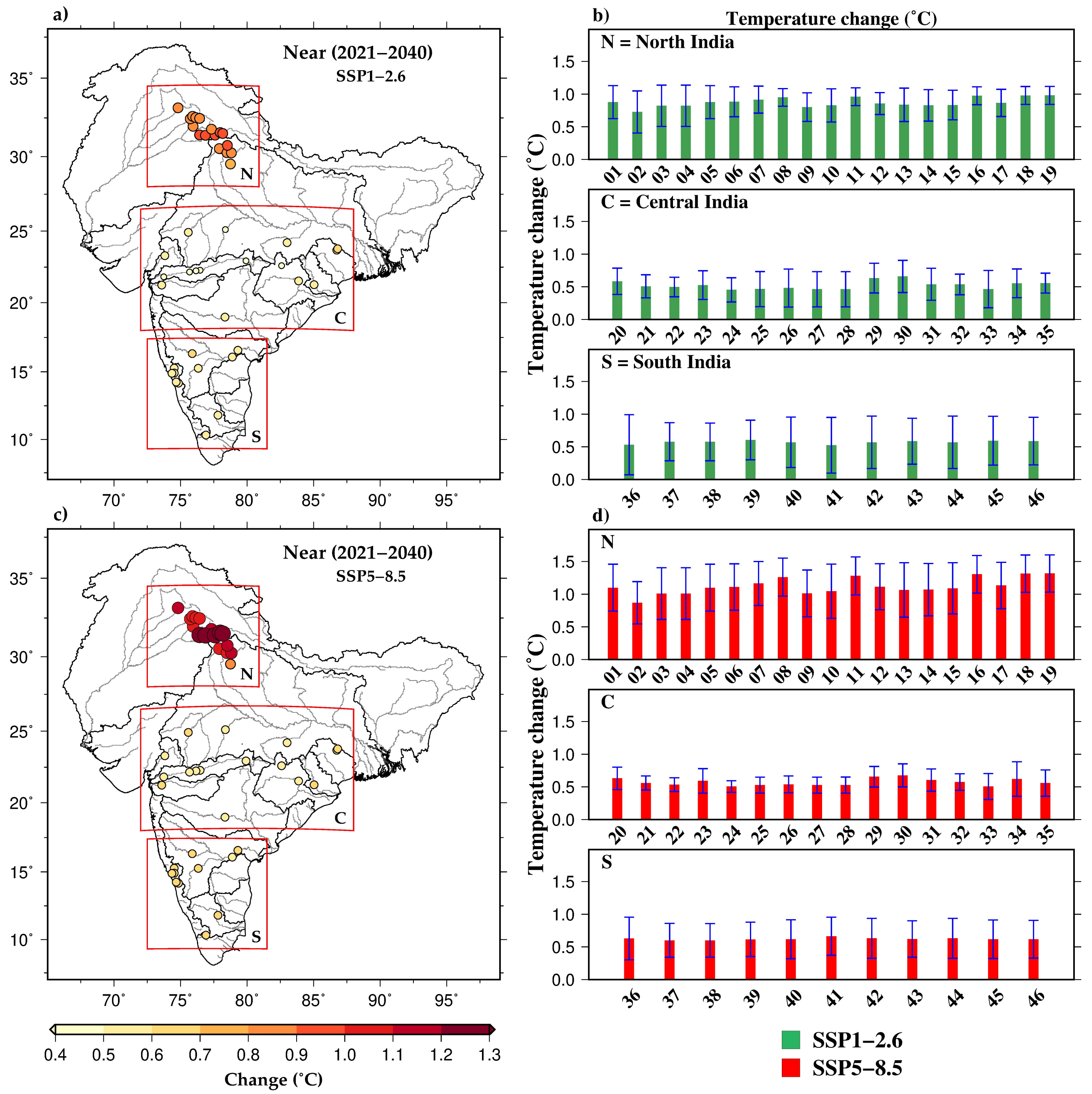

Figure S2: Multi-model mean changes in temperature (ºC) during the near period (2021-2040) compared to the historical reference period (1995-2014) for selected dams. Changes were calculated separately for SSP1-2.6 and SSP5-8.5 by averaging the changes for each GCM. The intermodel variation among 5 GCMs was represented by one standard deviation (blue ticks), Related to STAR Methods

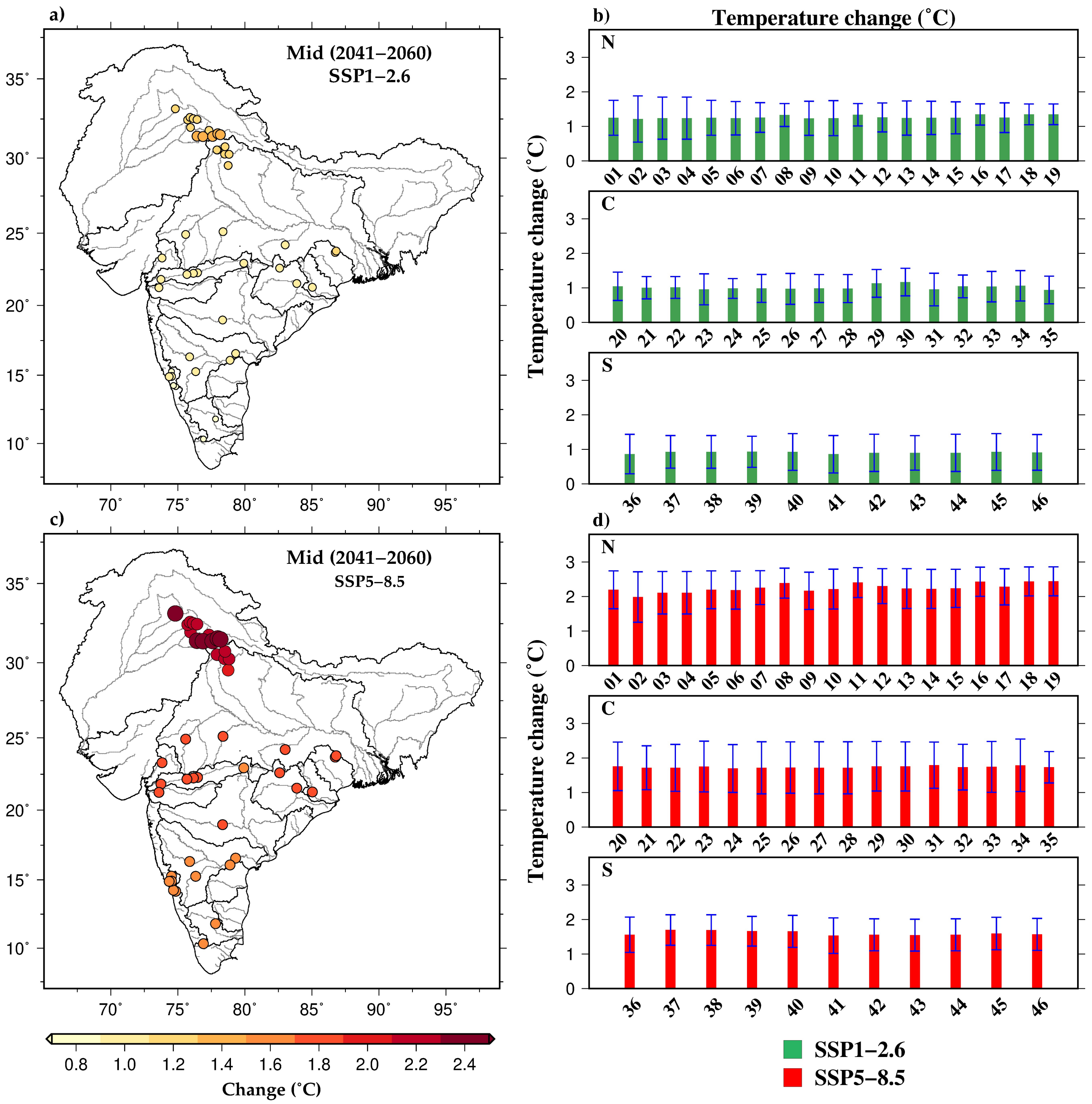

Figure S3: Multi-model mean changes in temperature (⁰C) during the mid period (2041-2060) compared to the historical reference period (1995-2014) for selected dams. Changes were calculated separately for SSP1-2.6 and SSP5-8.5 by averaging the changes for each GCM. The intermodel variation among 5 GCMs was represented by one standard deviation (blue ticks), Related to STAR Methods

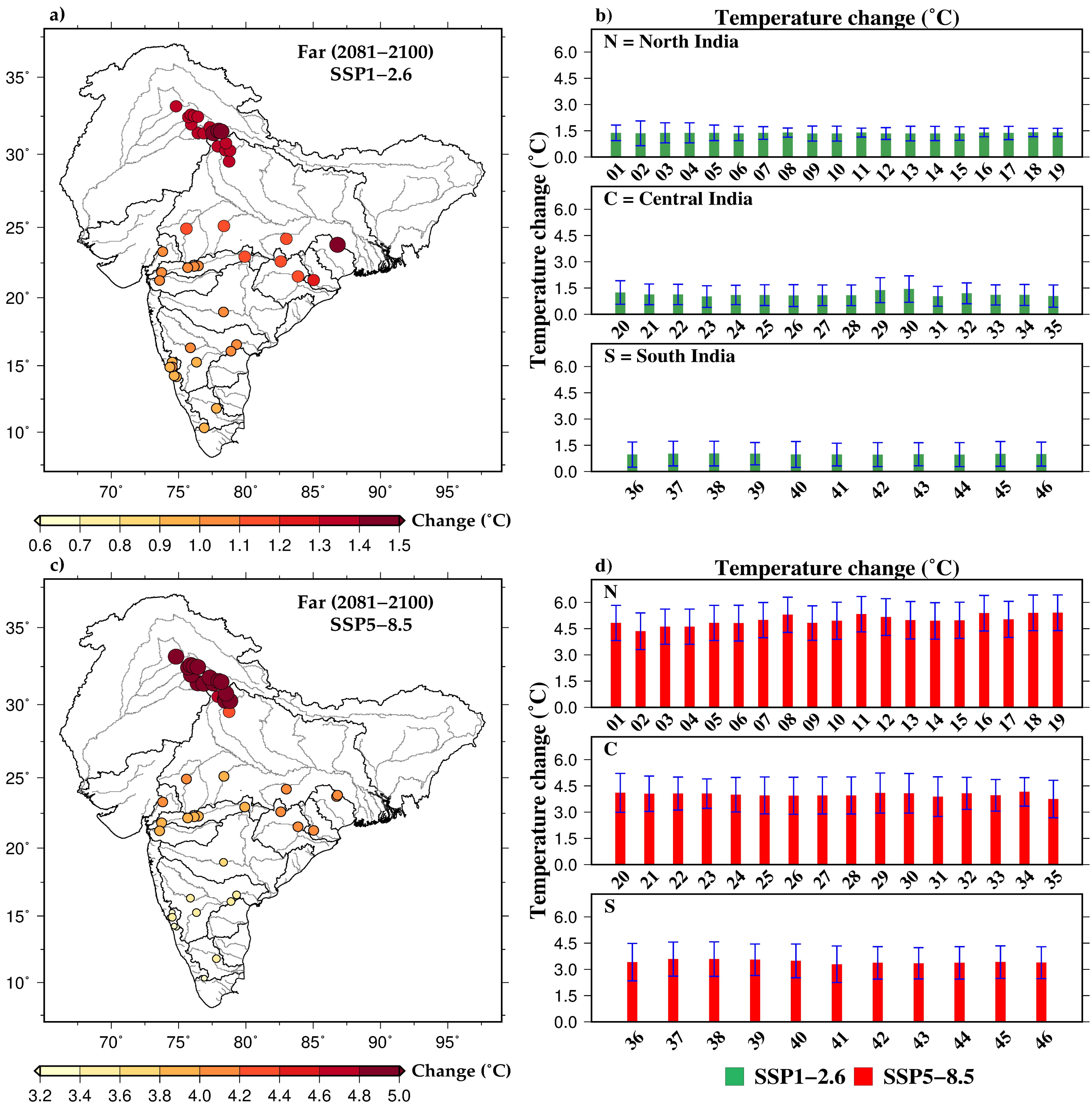

Figure S4: Multi-model mean changes in temperature (ºC) during the far period (2081-2100) compared to the historical reference period (1995-2014) for selected dams. Changes were calculated separately for SSP1-2.6 and SSP5-8.5 by averaging the changes for each GCM. The intermodel variation among 5 GCMs was represented by one standard deviation (blue ticks), Related to STAR Methods

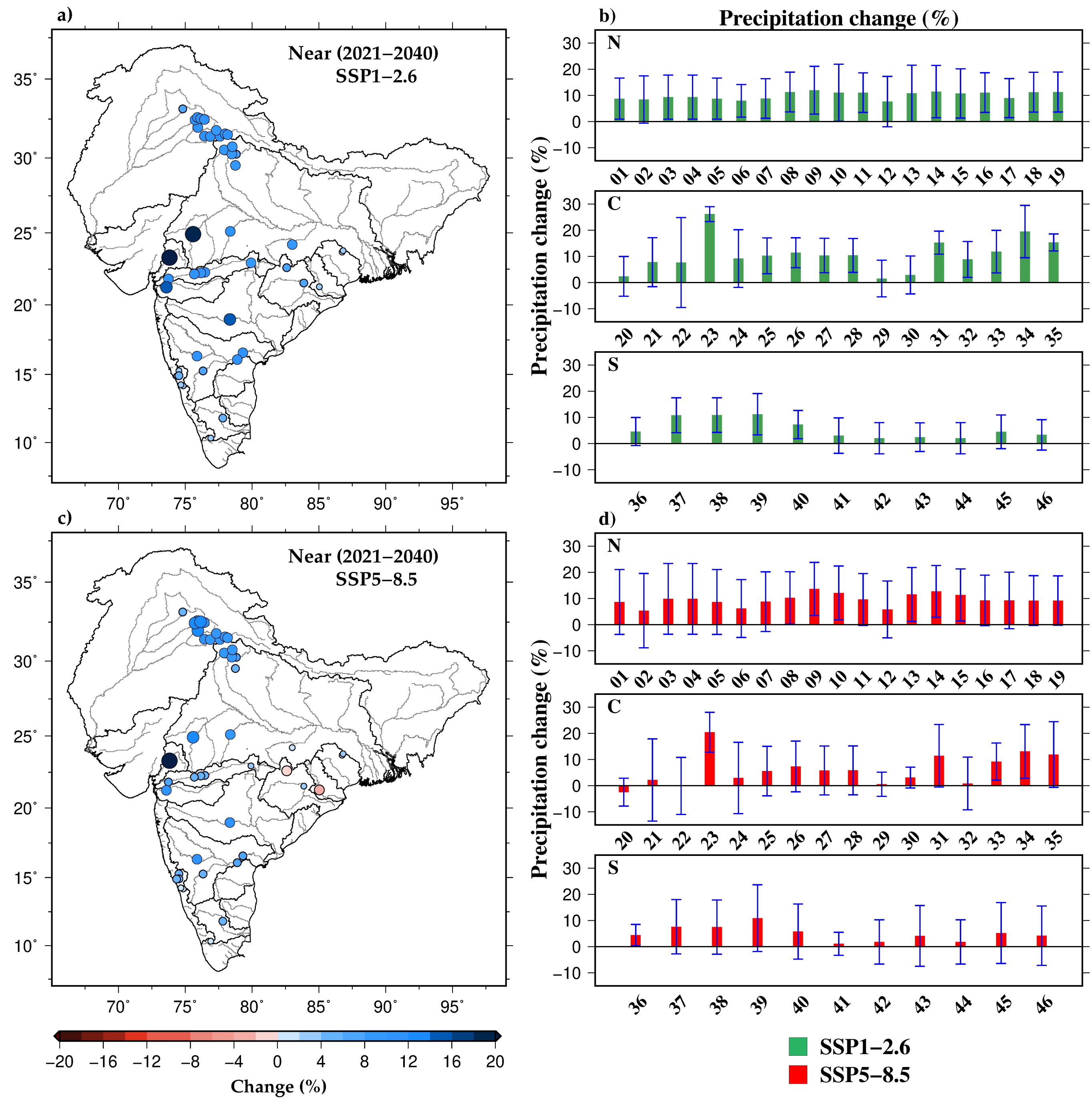

Figure S5: Multi-model mean changes in yearly precipitation (%) during the near period (2021-2040) compared to the historical reference period (1995-2014) for selected dams. Changes were calculated separately for SSP1-2.6 and SSP5-8.5 by averaging the changes for each GCM. The intermodel variation among 5 GCMs was represented by one standard deviation (blue ticks), Related to STAR Methods

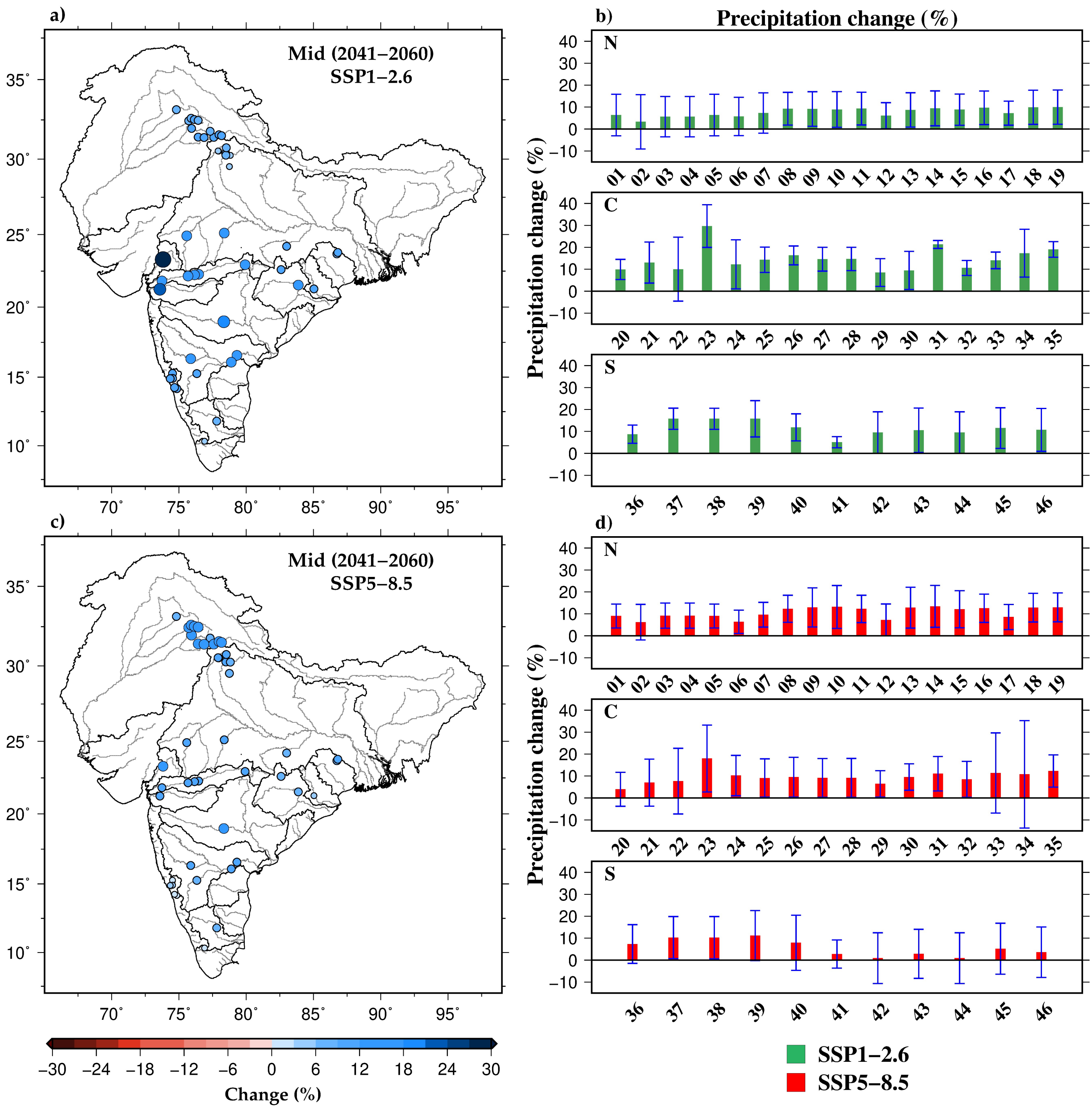

Figure S6: Multi-model mean changes in yearly precipitation (%) during the mid period (2041-2060) compared to the historical reference period (1995-2014) for selected dams. Changes were calculated separately for SSP1-2.6 and SSP5-8.5 by averaging the changes for each GCM. The intermodel variation among 5 GCMs was represented by one standard deviation (blue ticks), Related to STAR Methods

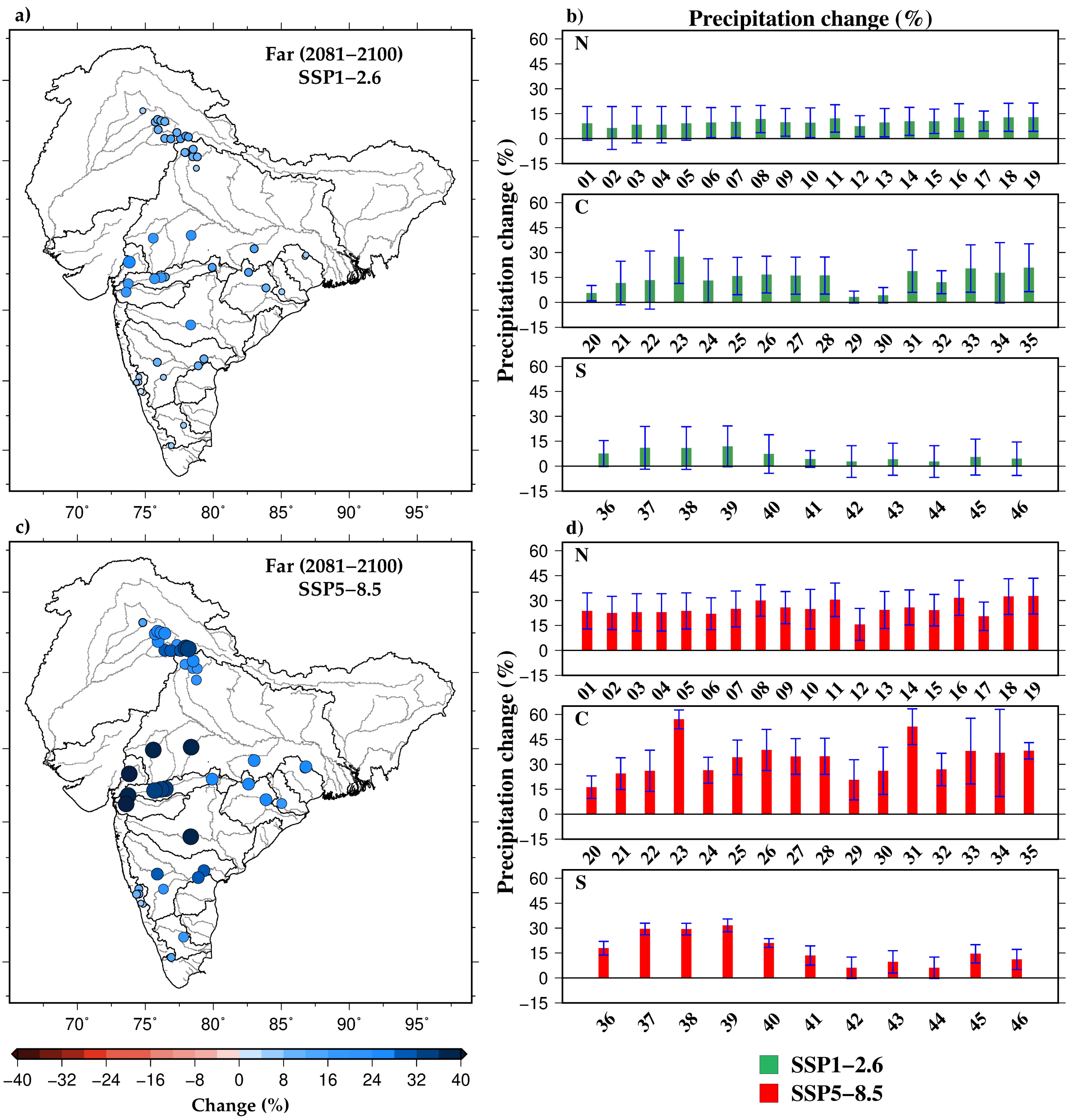

Figure S7: Multi-model mean changes in yearly precipitation (%) during the far period (2081-2100) compared to the historical reference period (1995-2014) for selected dams. Changes were calculated separately for SSP1-2.6 and SSP5-8.5 by averaging the changes for each GCM. The intermodel variation among 5 GCMs was represented by one standard deviation (blue ticks), Related to STAR Methods

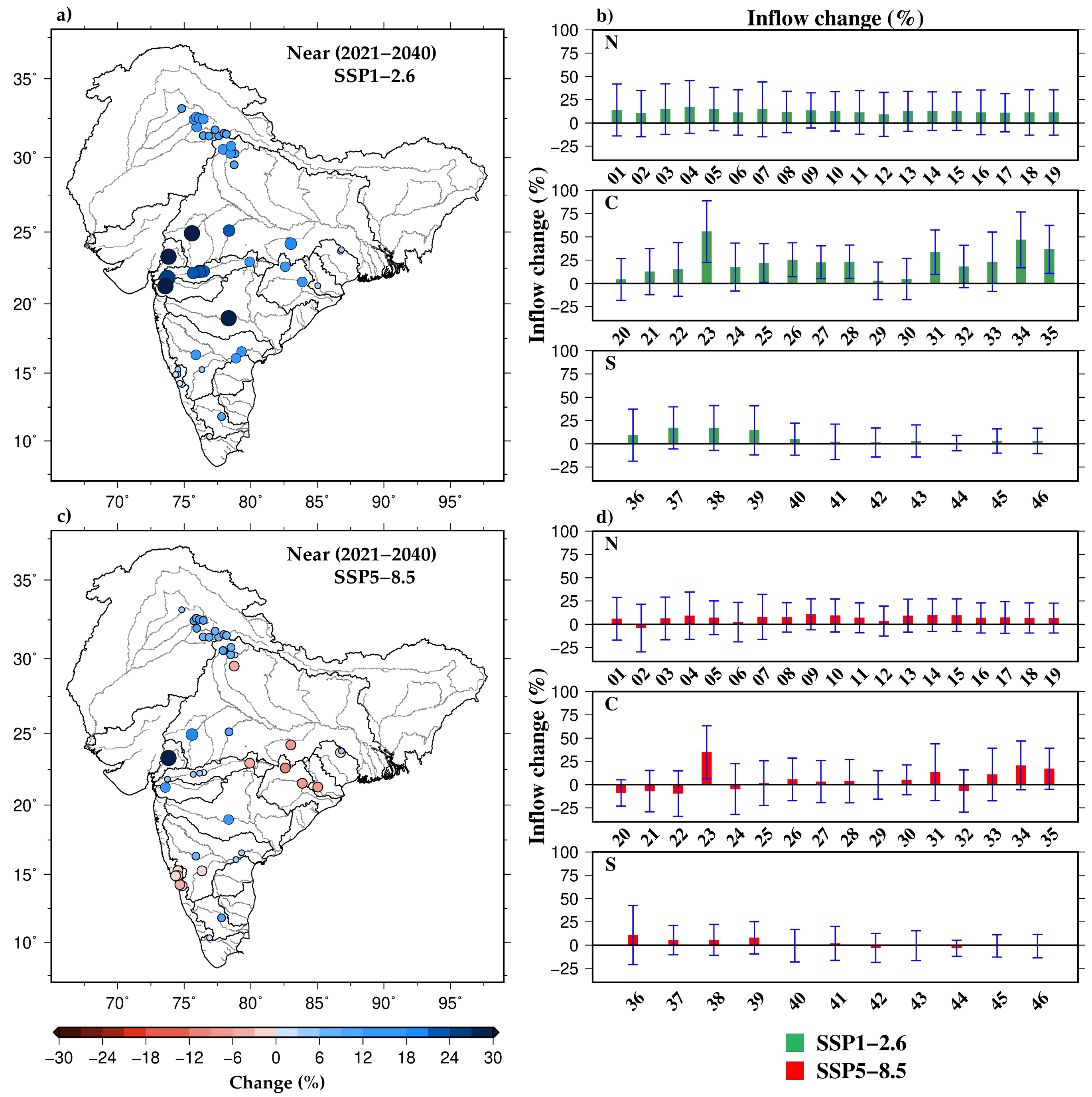

Figure S8: Multi-model mean changes in streamflow (%) during the near period (2021-2040) compared to the historical reference period (1995-2014) for selected dams. Changes were calculated separately for SSP1-2.6 and SSP5-8.5 by averaging the changes for each GCM. The intermodel variation among 5 GCMs was represented by one standard deviation (blue ticks), Related to Figure 3

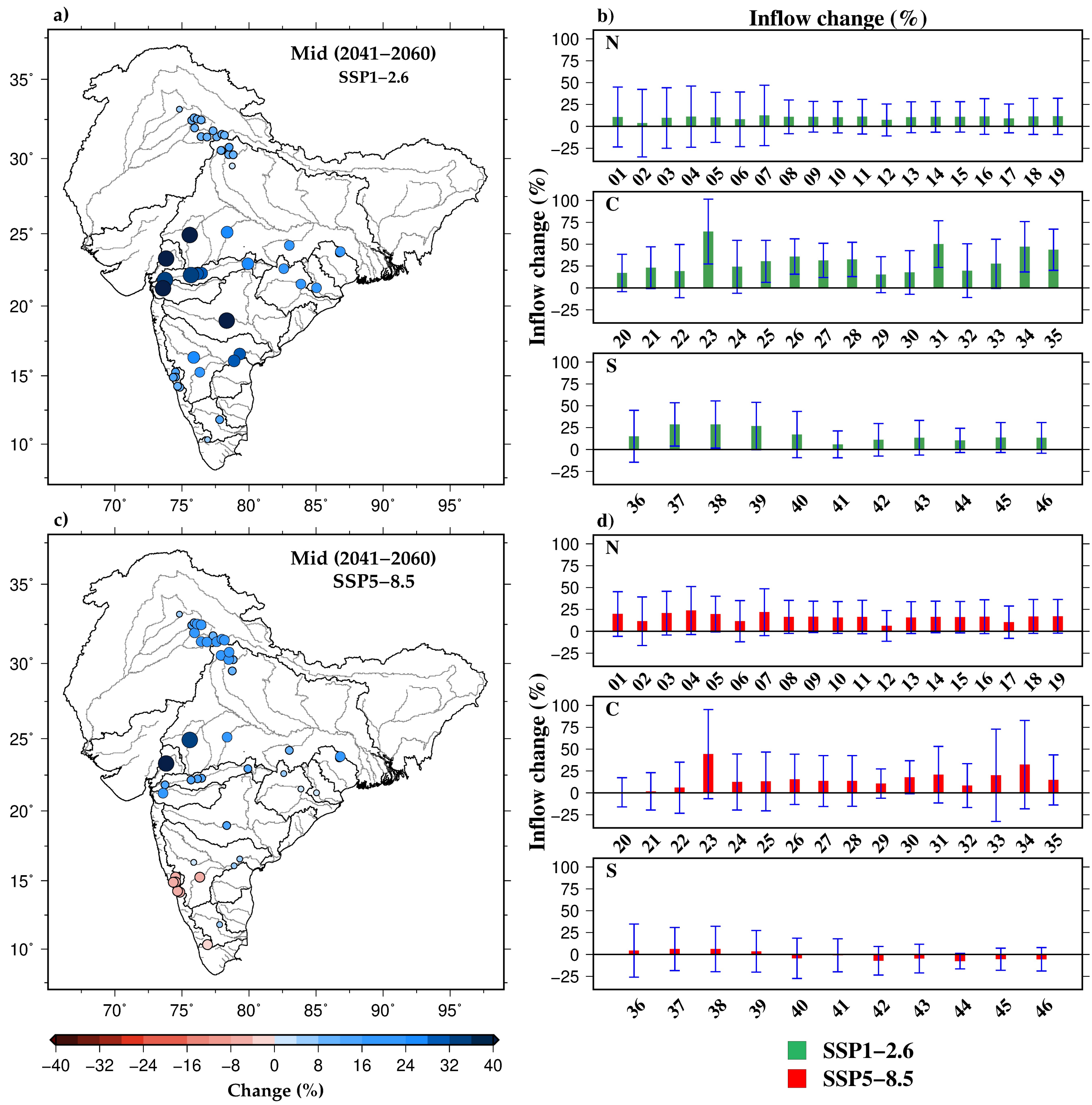

Figure S9: Multi-model mean changes in streamflow (%) during the mid period (2041-2060) compared to the historical reference period (1995-2014) for selected dams. Changes were calculated separately for SSP1-2.6 and SSP5-8.5 by averaging the changes for each GCM. The intermodel variation among 5 GCMs was represented by one standard deviation (blue ticks), Related to Figure 3

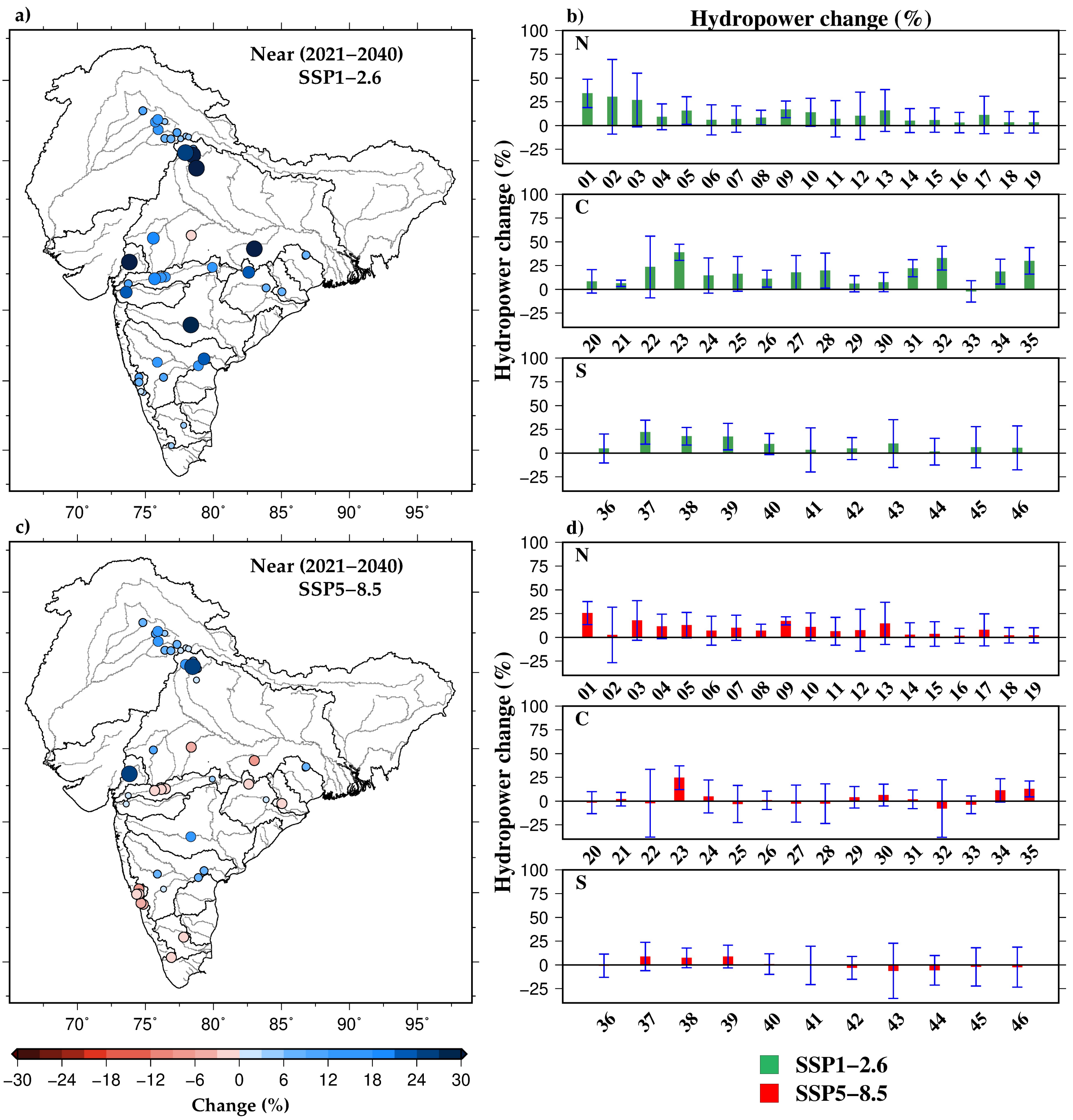

Figure S10: Multi-model mean changes in hydropower (%) during the near period (2021-2040) compared to the historical reference period (1995-2014) for selected dams. Changes were calculated separately for SSP1-2.6 and SSP5-8.5 by averaging the changes for each GCM. The intermodel variation among 5 GCMs was represented by one standard deviation (blue ticks), Related to Figure 4

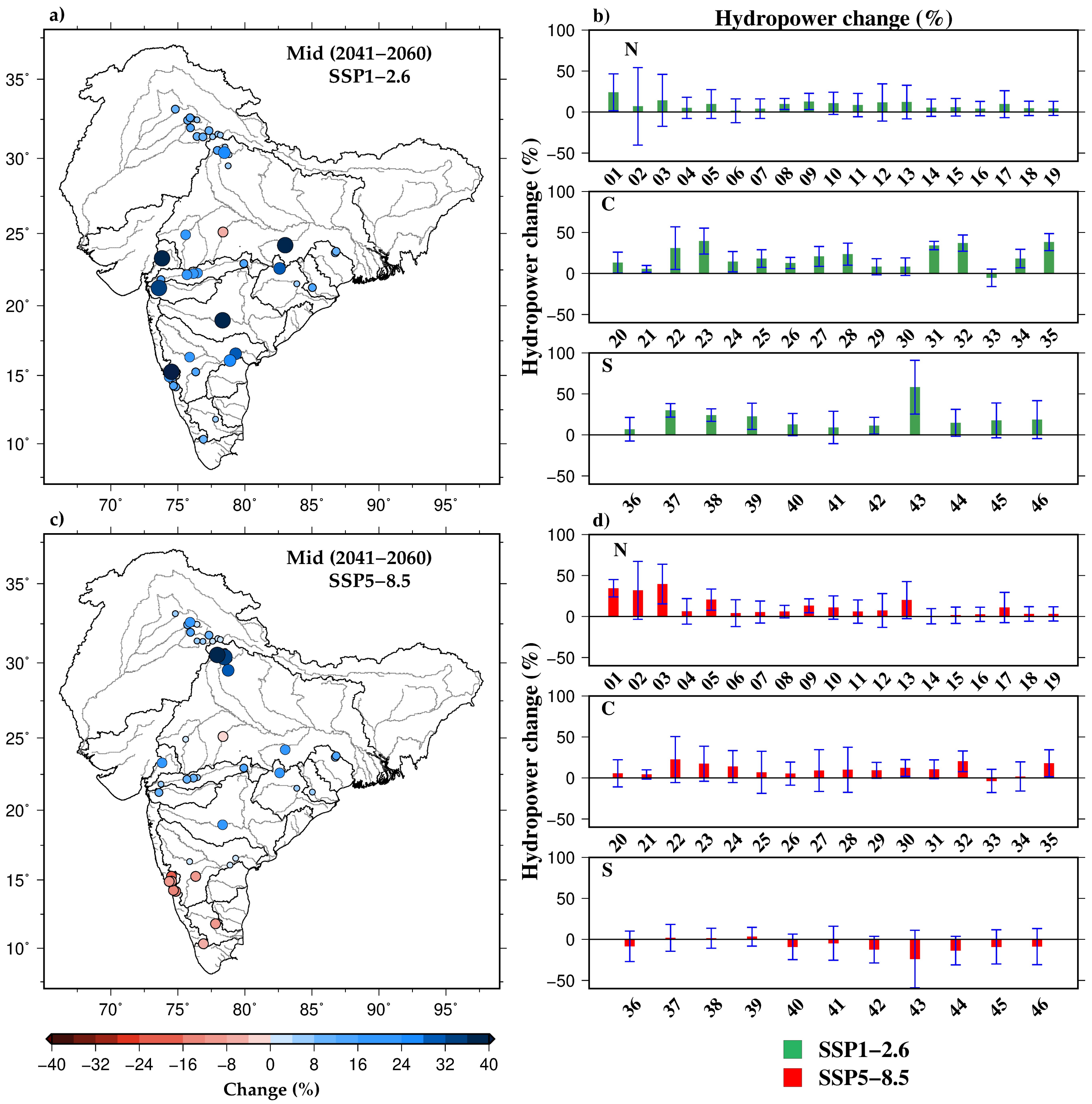

Figure S11: Multi-model mean changes in hydropower (%) during the mid period (2041-2060) compared to the historical reference period (1995-2014) for selected dams. Changes were calculated separately for SSP1-2.6 and SSP5-8.5 by averaging the changes for each GCM. The intermodel variation among 5 GCMs was represented by one standard deviation (blue ticks), Related to Figure 4

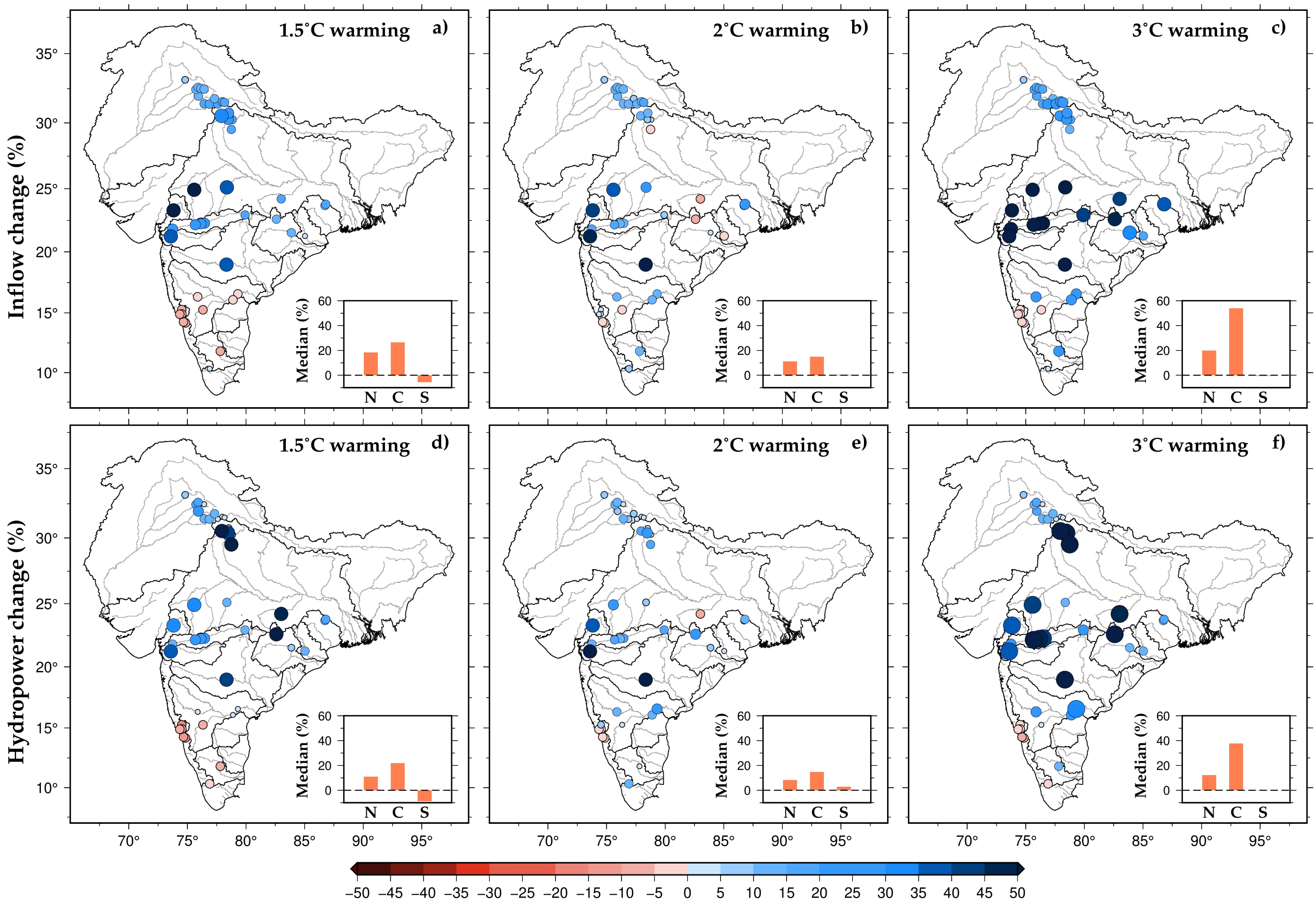

Figure S12: Percentage variation in streamflow, and hydropower potential for the selected dams under the selected global warming levels. Changes were computed compared to the historical reference period (1995-2014). Median change (%) in each region was represented as bars for each warming level, Related to STAR Methods

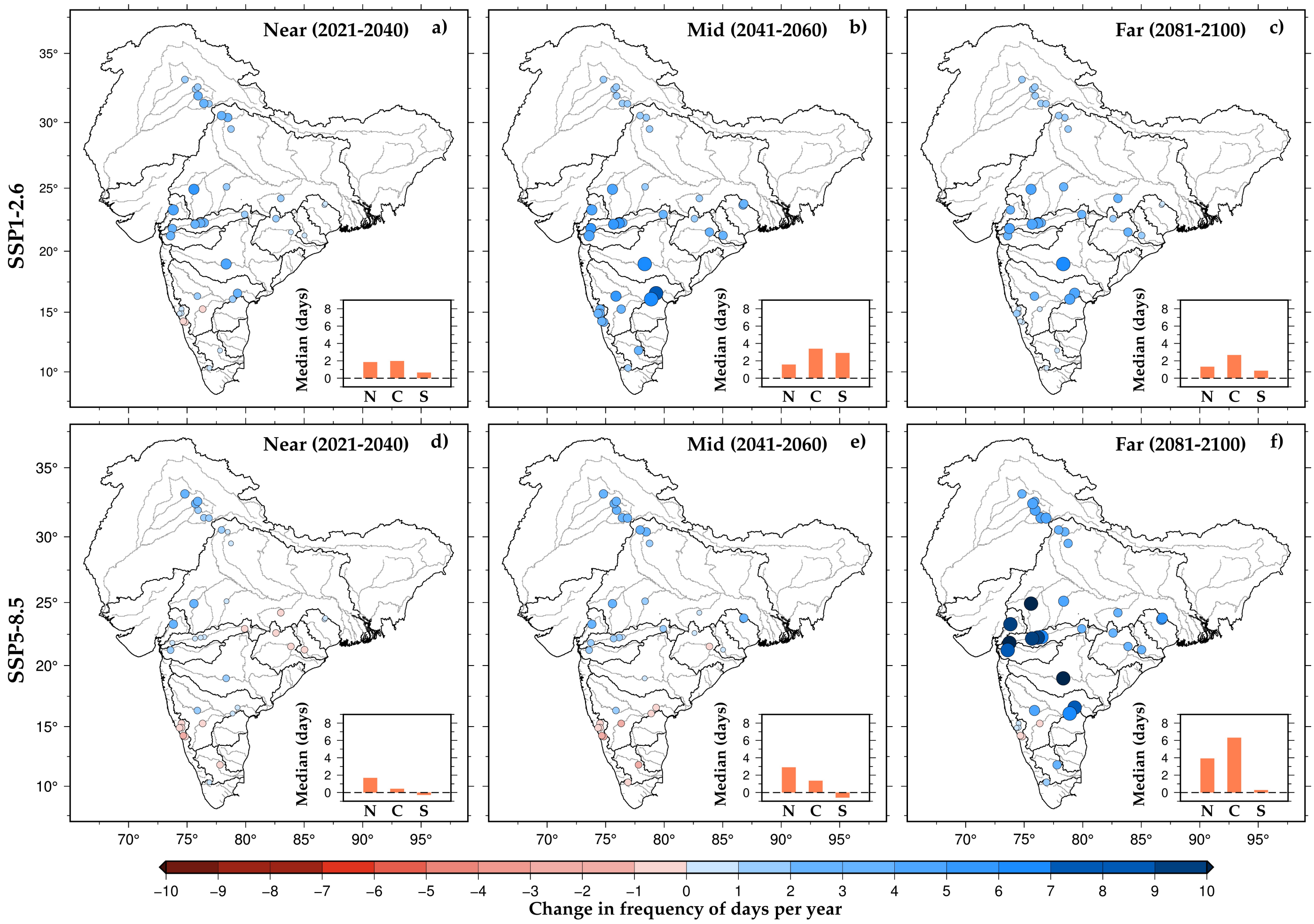

Figure S13: Multimodel mean change in the frequency of days (high flow events: inflow > 95th percentile) per year for near, mid, and far periods compared to the historical reference period. Median change (days) in each region was represented as bars for each period and under both SSPs, Related to Figure 5

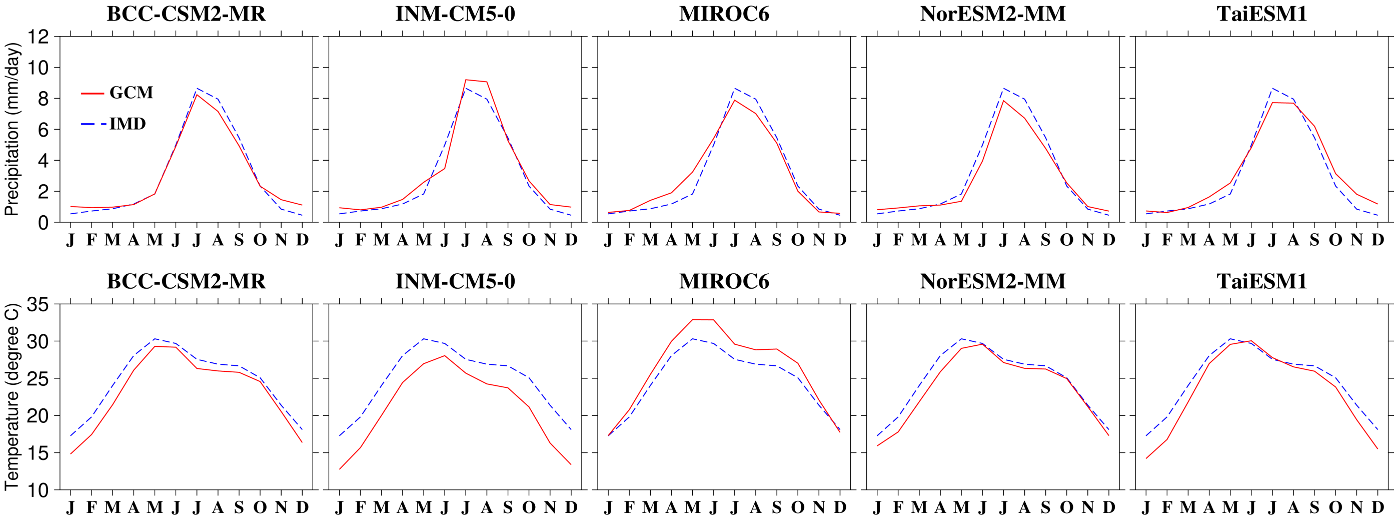

Figure S14: Comparison of seasonal cycle of average precipitation of selected GCMs and IMD over the Indian region before bias correction, Related to STAR Methods

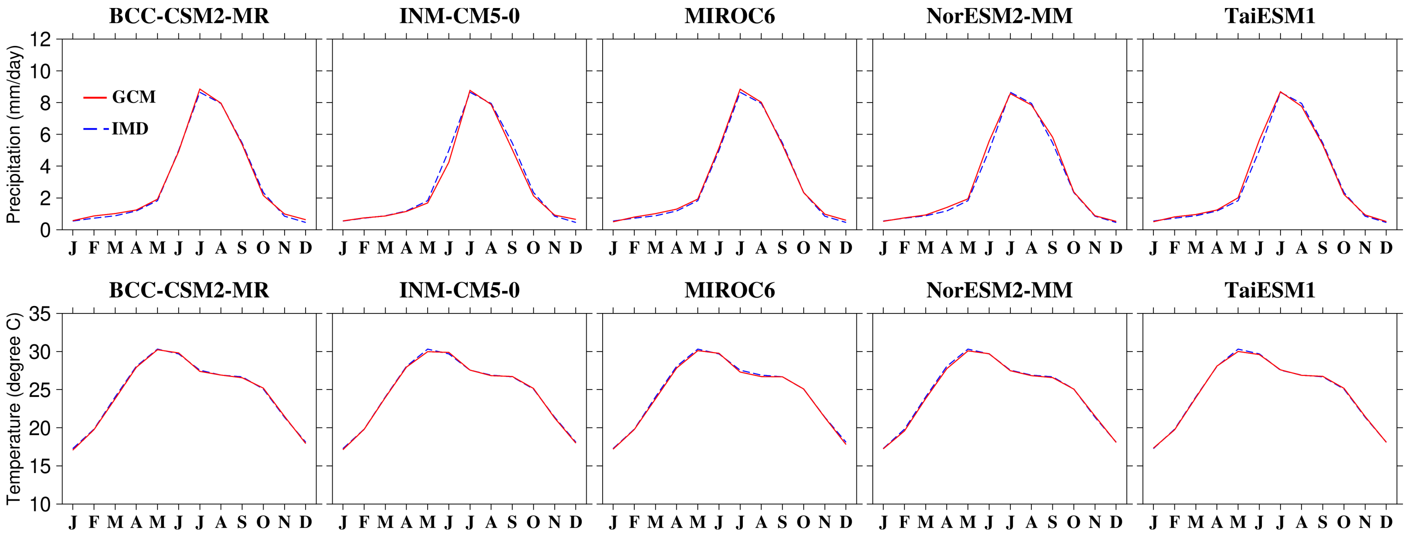

Figure S15: Comparison of seasonal cycle of average precipitation and temperature of selected GCMs and IMD over the Indian region after bias correction, Related to STAR Methods

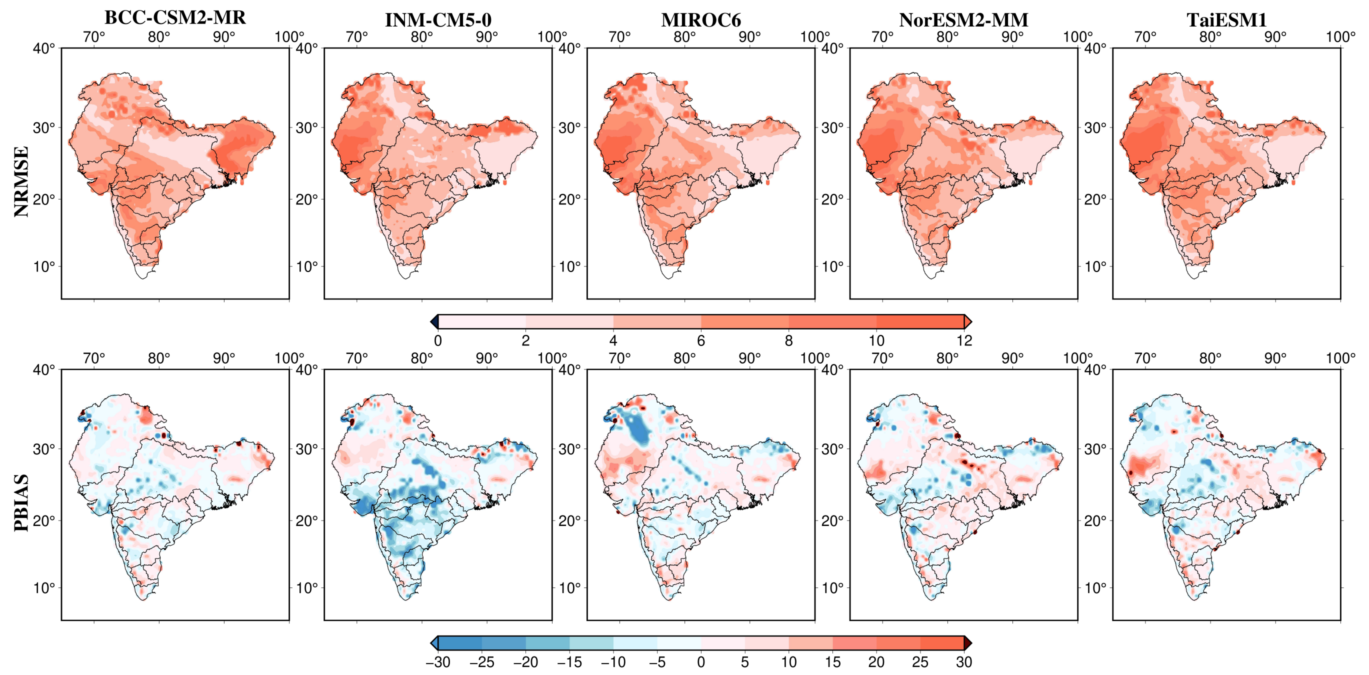

Figure S16: The spatial distribution of normalized root mean square index (NRMSE) and percentage bias (PBIAS) between the long-term historical mean precipitation (1951-2014) of CMIP6 data and observed precipitation after bias correction, Related to STAR Methods

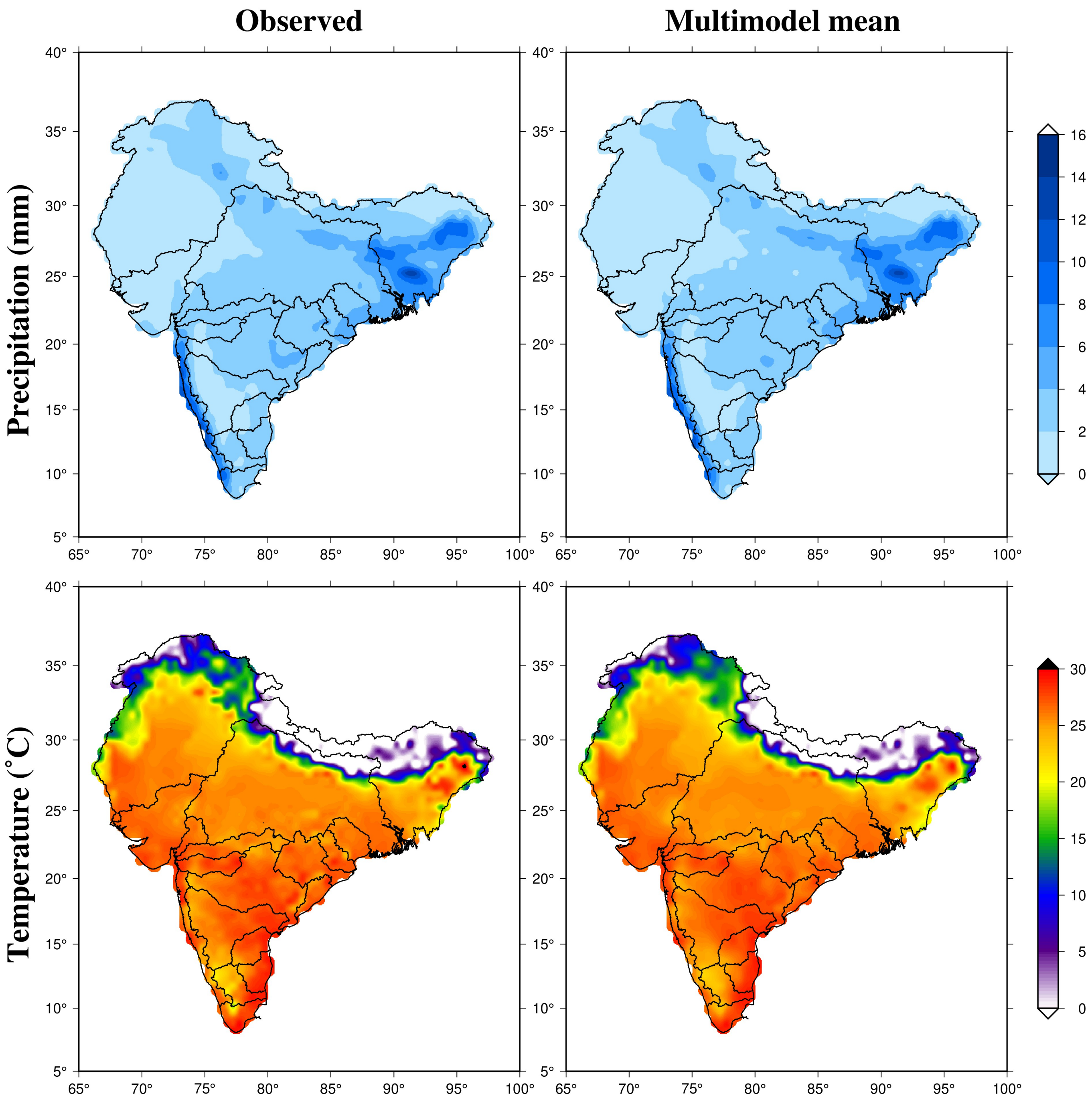

Figure S17: Long-term historical mean (1951-2014) spatial variation of observed and bias-corrected CMIP6 multi-model ensemble mean precipitation (mm) and temperature (⁰C) for ISC river basins, Related to STAR Methods

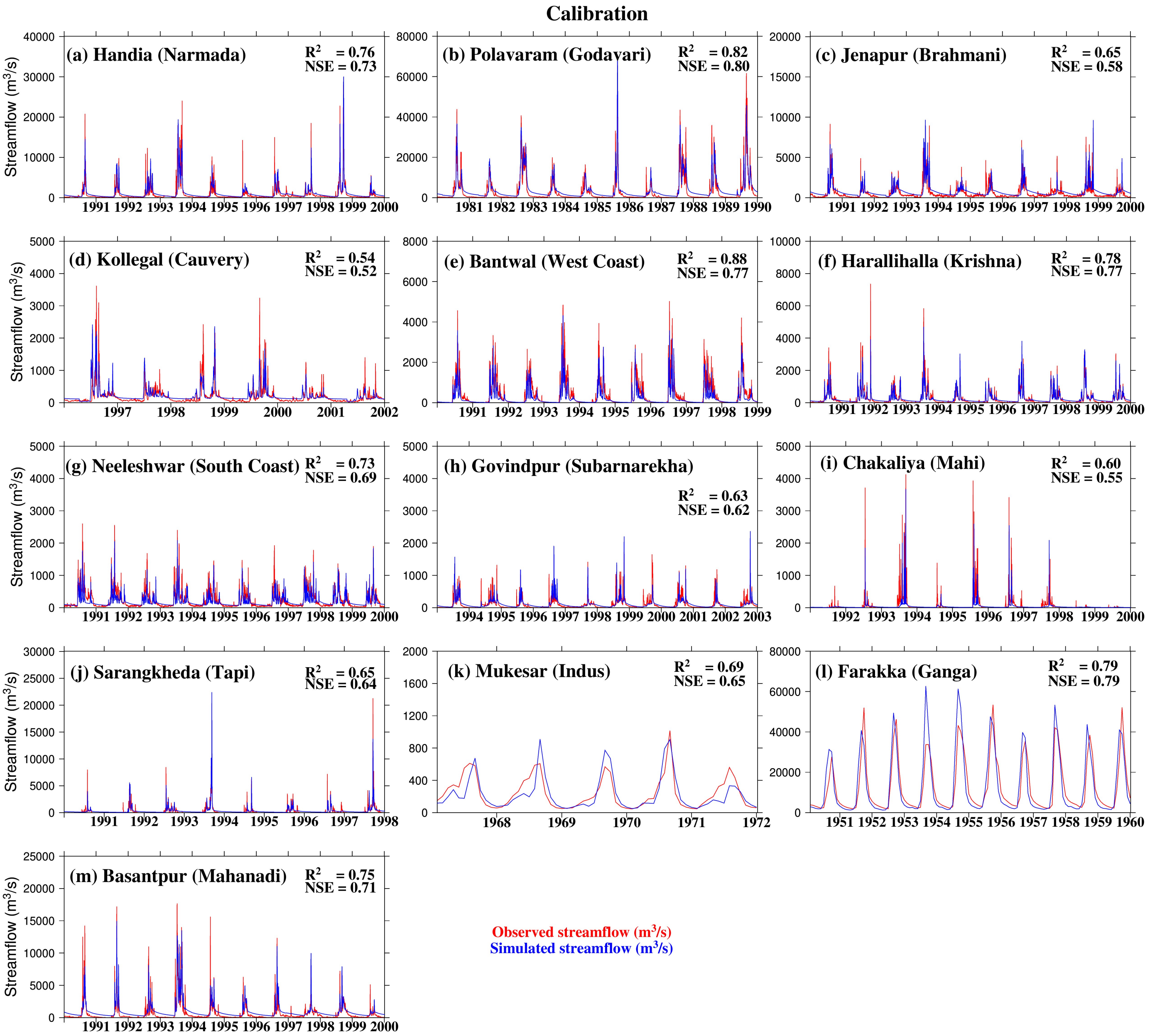

Figure S18: Calibration of the model against observed streamflow for 13 river basins. Ganga and Indus river basins were calibrated against monthly streamflow due to the unavailability of daily observed data. Nash Sutcliffe Efficiency (NSE) and coefficient of determination (R^2) were used to estimate the model performance, Related to STAR Methods

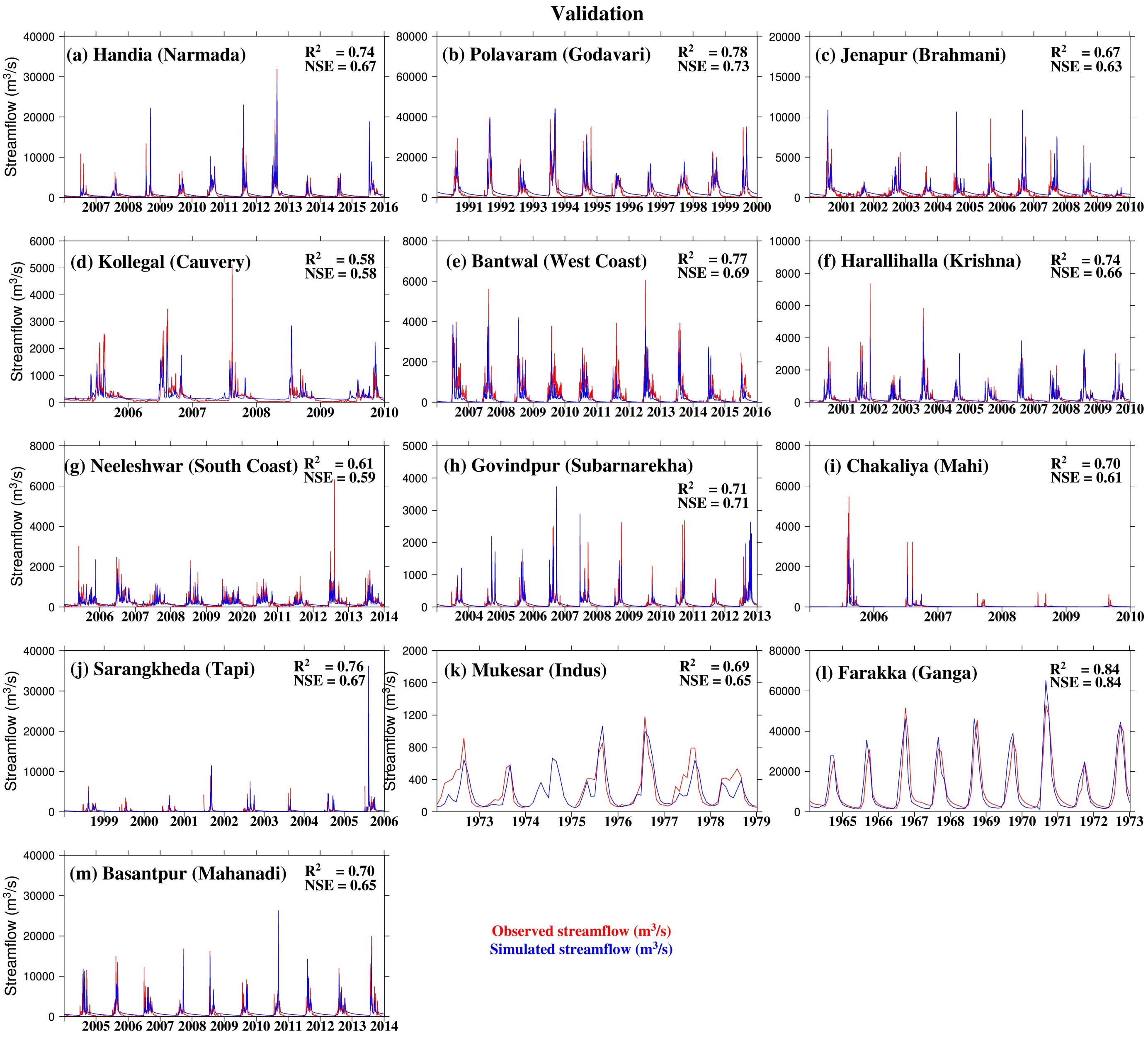

Figure S19: Validation of model against observed streamflow for 13 river basins. Ganga and Indus river basins were validated against monthly streamflow due to the unavailability of daily observed data. Nash Sutcliffe Efficiency (NSE) and coefficient of determination (R^2) were used to estimate the model performance, Related to STAR Methods

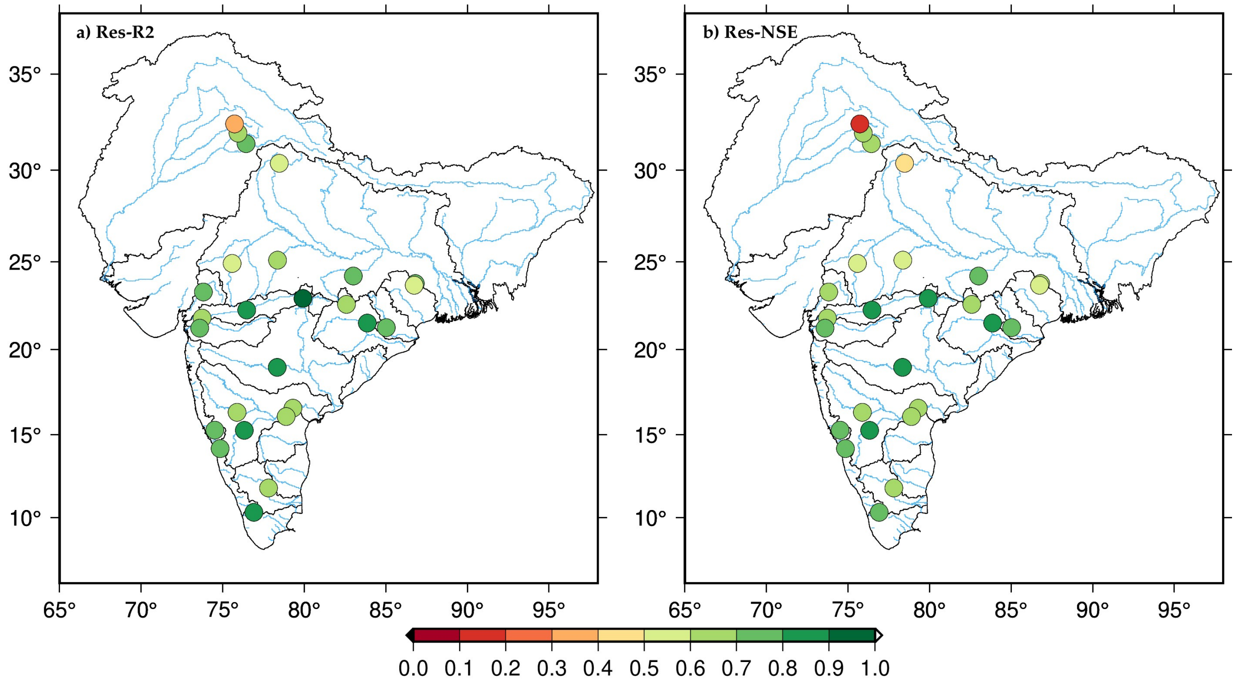

Figure S20: (a) Coefficient of determination (R^2) and (b) Nash Sutcliffe Efficiency (NSE) between observed reservoir storage and CaMa-Flood simulated reservoir storage based on observed meteorological forcing for 26 hydroelectric dams. The NSE and R^2 between observed and model-simulated reservoir storage for the rest 10 dams is not calculated due to the unavailability of observed reservoir storage data, Related to STAR Methods

Table S1: Details of the hydroelectricity producing dams (Source – Taken from NRLD and India WRIS), Related to STAR Methods

Storage type powerplants (S), Run-of-river powerplants (R)								
**Dam no.**	**Dam**	**Latitude**	**Longitude**	**Height or Net elevation (m)**	**Gross Storage Capacity (BCM)**	**Total Installed Capacity (MW)**	**Basin**	**Type**
**1**	TEHRI	30.37	78.47	260.50	3.54	1000	Ganga	S
**2**	RAMGANGA	29.51	78.75	128.00	2.45	198	Ganga	S
**3**	LAKHWAR	30.51	77.94	204.00	0.58	300	Ganga	S
**4**	VYASI	30.52	77.91	60	-	120	Ganga	R
**5**	KOTESHWAR	30.25	78.49	75	-	400	Ganga	R
**6**	SRINAGAR	30.24	78.82	66	-	330	Ganga	R
**7**	MANERIBHALI2	30.73	78.52	285	-	304	Ganga	R
**8**	BHAKRA	31.41	76.43	225.55	7.55	1325	Indus	S
**9**	PONG	31.96	75.94	132.59	9.62	396	Indus	S
**10**	THEIN	31.96	75.94	160.00	2.89	600	Indus	S
**11**	KOL	31.38	76.87	167.00	0.58	800	Indus	S
**12**	SALAL	33.14	74.81	113.00	0.27	690	Indus	S
**13**	CHAMERA1	32.60	75.91	121.00	0.39	540	Indus	S
**14**	CHAMERA2	32.52	76.14	243	-	300	Indus	R
**15**	CHAMERA3	32.47	76.43	200	-	231	Indus	R
**16**	RAMPUR	31.39	77.59	119	-	412	Indus	R
**17**	PARBATI2	31.78	77.32	863	-	800	Indus	R
**18**	NAPTHA	31.56	77.98	428	-	1500	Indus	R
**19**	KARCHAM	31.499	78.17	298	-	1000	Indus	R
**20**	RENGALI	21.27	85.03	70.50	5.15	250	Brahmani	S
**21**	HIRAKUD	21.53	83.87	60.96	8.14	347.5	Mahanadi	S
**22**	BANGO	22.60	82.59	87.00	3.42	120	Mahanadi	S
**23**	KADANA	23.30	73.82	66.00	1.54	240	Mahi	S
**24**	BARGI	22.94	79.92	69.80	3.92	105	Narmada	S
**25**	INDIRASAGAR	22.28	76.47	91.40	12.22	1000	Narmada	S
**26**	SARDARSAROVAR	21.82	73.74	163.00	1.76	1450	Narmada	S
**27**	OMKARESHWAR	22.24	76.16	64.00	0.99	525	Narmada	S
**28**	MAHESHWAR	22.16	75.68	36.00	0.48	400	Narmada	S
**29**	PANCHET	23.68	86.74	56.08	0.28	80	Subarnarekha	S
**30**	MAITHON	23.78	86.81	45.00	0.53	60	Subarnarekha	S
**31**	UKAI	21.24	73.59	80.77	7.50	300	Tapi	S
**32**	RIHAND	24.20	83.01	91.46	10.60	300	Ganga	S
**33**	MATATILA	25.10	78.37	45.72	1.13	33	Ganga	S
**34**	RANAPRATAP	24.91	75.58	53.80	2.89	172	Ganga	S
**35**	SRIRAMSAGAR	18.96	78.34	43.00	3.17	43	Godavari	S

**36**	METTUR	11.80	77.81	70.41	2.71	240	Cauvery	S
**37**	NAGARJUN	16.57	79.31	124.66	11.60	816	Krishna	S
**38**	SRISAILAM	16.08	78.89	145.00	8.72	1670	Krishna	S
**39**	ALMATTI	16.33	75.88	49.29	3.49	290	Krishna	S
**40**	TUNGABHADRA	15.26	76.33	49.39	3.16	127	Krishna	S
**41**	SHOLAYAR	10.33	76.91	66.14	0.15	120	South-Coast	S
**42**	LINGANAMAKKI	14.17	74.84	61.26	4.44	55	West-Coast	S
**43**	SUPA	15.27	74.52	101.00	4.18	100	West-Coast	S
**44**	GERUSOPA	14.25	74.67	62.00	0.13	240	West-Coast	S
**45**	KODASALLI	14.91	74.53	52.10	0.29	120	West-Coast	S
**46**	KADRA	14.89	74.35	40.50	0.39	150	West-Coast	S

Table S2: Multi-model mean changes and inter-model uncertainty (standard deviation) in temperature under SSP1-2.6, SSP5-8.5 for the Near (2021-2040), Mid (2041-2060), and Far (2081-2100) terms, Related to STAR Methods

Temperature change (ºC)												
Dam	Near period				Mid period				Far period			
	SSP1-2.6		SSP5-8.5		SSP1-2.6		SSP5-8.5		SSP1-2.6		SSP5-8.5	
	Mean	Std	Mean	Std	Mean	Std	Mean	Std	Mean	Std	Mean	Std
TEHRI	0.88	0.25	1.10	0.36	1.25	0.51	2.19	0.55	1.38	0.45	4.83	1.00
RAMGANGA	0.73	0.32	0.87	0.32	1.21	0.67	1.99	0.73	1.35	0.71	4.35	1.05
LAKHWAR	0.82	0.32	1.01	0.40	1.24	0.61	2.11	0.62	1.38	0.57	4.61	1.00
VYASI	0.82	0.32	1.01	0.40	1.24	0.61	2.11	0.62	1.38	0.57	4.61	1.00
KOTESHWAR	0.88	0.25	1.10	0.36	1.25	0.51	2.19	0.55	1.38	0.45	4.83	1.00
SRINAGAR	0.88	0.23	1.11	0.36	1.24	0.49	2.19	0.55	1.34	0.41	4.82	1.02
MANERIBHALI2	0.92	0.21	1.16	0.34	1.26	0.43	2.26	0.49	1.38	0.36	4.99	1.00
BHAKRA	0.95	0.14	1.26	0.29	1.33	0.33	2.39	0.44	1.40	0.26	5.29	1.00
PONG	0.80	0.22	1.01	0.36	1.23	0.49	2.17	0.54	1.34	0.43	4.82	0.98
THEIN	0.83	0.26	1.05	0.42	1.24	0.51	2.22	0.58	1.34	0.43	4.95	1.06
KOL	0.96	0.14	1.28	0.29	1.34	0.32	2.41	0.43	1.40	0.26	5.33	1.01
SALAL	0.86	0.17	1.11	0.35	1.26	0.42	2.30	0.51	1.34	0.34	5.16	1.05
CHAMERA1	0.84	0.26	1.06	0.42	1.24	0.50	2.23	0.58	1.34	0.42	4.98	1.07
CHAMERA2	0.83	0.24	1.07	0.40	1.25	0.48	2.22	0.57	1.34	0.41	4.94	1.04
CHAMERA3	0.83	0.23	1.09	0.39	1.25	0.46	2.24	0.55	1.34	0.38	4.98	1.04
RAMPUR	0.97	0.14	1.31	0.29	1.34	0.31	2.43	0.42	1.40	0.25	5.38	1.02
PARBATI2	0.87	0.21	1.13	0.35	1.25	0.43	2.28	0.52	1.38	0.38	5.03	1.03
NAPTHA	0.98	0.14	1.31	0.29	1.35	0.30	2.44	0.42	1.40	0.24	5.40	1.02
KARCHAM	0.98	0.14	1.32	0.29	1.35	0.30	2.44	0.42	1.40	0.24	5.41	1.02
RENGALI	0.58	0.20	0.63	0.17	1.05	0.41	1.76	0.70	1.24	0.67	4.10	1.11
HIRAKUD	0.51	0.18	0.56	0.11	1.00	0.32	1.72	0.64	1.13	0.59	4.05	1.01
BANGO	0.50	0.15	0.54	0.10	1.01	0.32	1.72	0.68	1.13	0.58	4.06	0.94
KADANA	0.53	0.22	0.59	0.19	0.96	0.45	1.75	0.74	1.01	0.62	4.06	0.84
BARGI	0.45	0.19	0.50	0.09	0.98	0.29	1.70	0.69	1.10	0.55	4.00	0.99
INDIRASAGAR	0.47	0.27	0.53	0.12	0.99	0.40	1.72	0.75	1.09	0.59	3.95	1.06
SARDARSAROVAR	0.48	0.29	0.54	0.13	0.97	0.45	1.72	0.75	1.07	0.62	3.94	1.06
OMKARESHWAR	0.46	0.27	0.53	0.12	0.98	0.41	1.72	0.75	1.09	0.59	3.95	1.06
MAHESHWAR	0.46	0.27	0.53	0.12	0.98	0.41	1.72	0.75	1.08	0.59	3.95	1.06
PANCHET	0.63	0.23	0.66	0.16	1.13	0.40	1.76	0.72	1.38	0.71	4.09	1.15
MAITHON	0.66	0.25	0.68	0.18	1.17	0.40	1.75	0.71	1.44	0.76	4.07	1.13
UKAI	0.54	0.25	0.60	0.17	0.95	0.47	1.79	0.67	1.03	0.57	3.89	1.13
RIHAND	0.54	0.16	0.57	0.13	1.04	0.33	1.74	0.66	1.20	0.60	4.07	0.92
MATATILA	0.46	0.29	0.51	0.20	1.04	0.44	1.74	0.74	1.10	0.57	3.96	0.90
RANAPRATAP	0.55	0.22	0.62	0.26	1.06	0.44	1.79	0.76	1.10	0.60	4.16	0.82
SRIRAMSAGAR	0.56	0.15	0.56	0.20	0.94	0.40	1.73	0.46	1.04	0.63	3.75	1.07
METTUR	0.53	0.46	0.63	0.33	0.86	0.57	1.56	0.51	0.97	0.72	3.41	1.07
NAGARJUN	0.58	0.29	0.60	0.26	0.93	0.47	1.70	0.44	1.02	0.71	3.59	0.98
SRISAILAM	0.58	0.29	0.60	0.26	0.93	0.47	1.69	0.44	1.03	0.71	3.59	0.98
ALMATTI	0.60	0.30	0.62	0.26	0.93	0.45	1.66	0.43	1.02	0.64	3.55	0.90
TUNGABHADRA	0.57	0.39	0.62	0.30	0.92	0.53	1.66	0.46	0.97	0.74	3.49	0.96
SHOLAYAR	0.52	0.43	0.66	0.29	0.86	0.54	1.53	0.52	0.97	0.65	3.29	1.04
LINGANAMAKKI	0.57	0.40	0.63	0.31	0.90	0.54	1.56	0.46	0.97	0.68	3.37	0.93
SUPA	0.59	0.35	0.62	0.28	0.90	0.50	1.55	0.46	0.98	0.66	3.35	0.90
GERUSOPA	0.57	0.40	0.63	0.31	0.90	0.54	1.56	0.46	0.97	0.68	3.37	0.93
KODASALLI	0.59	0.37	0.62	0.29	0.92	0.53	1.59	0.47	1.01	0.71	3.42	0.93
KADRA	0.59	0.36	0.62	0.29	0.91	0.52	1.57	0.46	0.99	0.69	3.38	0.91

Table S3: Multi-model mean changes and inter-model uncertainty (standard deviation) in precipitation under SSP1-2.6, SSP5-8.5 for the Near (2021-2040), Mid (2041-2060), and Far (2081-2100) terms, Related to STAR Methods

Precipitation change (%)												
**Dam**	**Near period**				**Mid period**				**Far period**			
	**SSP1-2.6**		**SSP5-8.5**		**SSP1-2.6**		**SSP5-8.5**		**SSP1-2.6**		**SSP5-8.5**	
	**Mean**	**Std**	**Mean**	**Std**	**Mean**	**Std**	**Mean**	**Std**	**Mean**	**Std**	**Mean**	**Std**
TEHRI	8.75	7.86	8.68	12.41	6.39	9.47	9.02	5.48	9.24	10.16	23.74	10.87
RAMGANGA	8.43	9.01	5.38	14.23	3.30	12.39	6.21	8.13	6.40	12.90	22.51	10.02
LAKHWAR	9.34	8.42	9.87	13.52	5.64	9.18	9.17	5.71	8.36	10.96	22.90	11.25
VYASI	9.34	8.42	9.87	13.52	5.64	9.18	9.17	5.71	8.36	10.96	22.90	11.25
KOTESHWAR	8.75	7.86	8.68	12.41	6.39	9.47	9.02	5.48	9.24	10.16	23.74	10.87
SRINAGAR	7.93	6.22	6.19	11.07	5.71	8.71	6.38	5.32	9.62	9.03	22.06	9.59
MANERIBHALI2	8.83	7.58	8.80	11.41	7.31	9.18	9.61	5.65	9.98	9.38	24.93	10.79
BHAKRA	11.29	7.60	10.23	10.02	9.26	7.45	12.32	6.15	11.76	8.19	30.04	9.48
PONG	11.99	9.14	13.68	10.15	9.12	7.87	12.92	8.90	9.84	8.32	25.79	9.67
THEIN	11.07	10.89	12.14	10.28	8.91	8.11	13.16	9.76	9.55	8.97	24.81	11.92
KOL	11.06	7.56	9.65	9.93	9.33	7.47	12.23	6.23	12.13	8.26	30.47	10.05
SALAL	7.63	9.65	5.84	10.89	6.08	5.95	7.21	7.30	7.49	6.32	15.64	9.61
CHAMERA1	10.78	10.76	11.55	10.29	8.68	7.82	12.82	9.29	9.61	8.55	24.32	11.15
CHAMERA2	11.46	10.00	12.74	9.92	9.42	7.94	13.41	9.54	10.42	8.42	25.85	10.54
CHAMERA3	10.77	9.40	11.38	9.95	8.82	7.10	12.09	8.45	10.40	7.33	24.20	9.52
RAMPUR	11.09	7.58	9.27	9.66	9.70	7.65	12.53	6.44	12.65	8.36	31.64	10.57
PARBATI2	8.97	7.44	9.28	10.80	7.23	5.48	8.54	5.71	10.54	6.01	20.54	8.52
NAPTHA	11.23	7.63	9.21	9.52	9.90	7.76	12.82	6.55	12.84	8.44	32.40	10.74
KARCHAM	11.30	7.64	9.22	9.46	9.97	7.80	12.93	6.58	12.90	8.49	32.66	10.77
RENGALI	2.38	7.61	-2.49	5.34	9.91	4.60	3.95	7.77	5.58	4.61	16.25	6.75
HIRAKUD	7.80	9.38	2.18	15.72	13.05	9.36	6.99	10.73	11.67	13.11	24.41	9.55
BANGO	7.65	17.20	-0.09	17.90	10.03	14.55	7.67	14.99	13.45	17.50	26.10	12.40
KADANA	26.18	7.86	20.41	7.66	29.65	12.72	17.98	19.22	27.43	16.00	57.04	22.67
BARGI	9.19	11.03	2.96	13.64	12.21	11.18	10.21	9.19	13.09	13.17	26.44	7.82
INDIRASAGAR	10.23	6.87	5.58	9.47	14.33	5.77	9.09	8.75	15.86	11.22	34.22	10.43
SARDARSAROVAR	11.43	5.71	7.35	9.68	16.31	4.30	9.46	9.07	16.73	11.08	38.61	12.37
OMKARESHWAR	10.36	6.60	5.81	9.38	14.58	5.43	9.15	8.83	16.09	11.14	34.69	10.77
MAHESHWAR	10.40	6.50	5.87	9.35	14.67	5.32	9.16	8.88	16.16	11.13	34.84	10.88
PANCHET	1.53	7.02	0.55	4.62	8.51	6.33	6.42	6.02	3.21	3.53	20.65	12.10
MAITHON	2.90	7.27	3.09	3.96	9.45	8.67	9.53	6.03	4.28	4.66	26.11	14.22
UKAI	15.26	4.41	11.45	11.97	21.31	1.81	11.04	7.89	18.79	12.77	52.63	10.72
RIHAND	8.86	6.88	0.84	10.06	10.58	3.39	8.51	8.19	12.15	6.87	26.88	9.77
MATATILA	11.84	8.15	9.20	7.09	14.03	3.78	11.38	18.27	20.35	14.29	37.96	19.78
RANAPRATAP	19.51	10.90	13.09	10.27	17.31	10.90	10.77	24.50	17.85	18.10	36.88	26.22
SRIRAMSAGAR	15.34	3.24	11.92	12.58	19.04	3.56	12.30	7.35	20.88	14.37	38.12	4.92
METTUR	4.61	5.37	4.44	4.06	8.71	4.14	7.34	8.83	7.59	7.79	17.88	4.13
NAGARJUN	10.83	6.69	7.62	10.37	15.77	4.85	10.29	9.60	11.01	12.90	29.49	3.49
SRISAILAM	10.90	6.63	7.50	10.36	15.74	4.81	10.22	9.63	10.91	12.86	29.36	3.52
ALMATTI	11.22	7.90	10.91	12.76	15.74	8.28	11.19	11.38	11.95	12.24	31.63	3.85
TUNGABHADRA	7.27	5.40	5.78	10.56	11.85	6.17	7.89	12.55	7.32	11.59	21.00	2.66
SHOLAYAR	3.05	6.79	1.10	4.40	5.09	2.55	2.78	6.42	4.30	5.05	13.49	5.77
LINGANAMAKKI	2.05	5.94	1.80	8.46	9.47	9.47	0.90	11.57	2.76	9.57	6.08	6.46
SUPA	2.44	5.49	4.13	11.63	10.51	10.12	2.88	11.17	4.17	9.61	9.65	6.74
GERUSOPA	2.05	5.94	1.80	8.46	9.47	9.47	0.90	11.57	2.76	9.57	6.08	6.46
KODASALLI	4.48	6.43	5.21	11.66	11.54	9.24	5.19	11.62	5.47	10.84	14.54	5.52
KADRA	3.30	5.81	4.19	11.34	10.69	9.77	3.61	11.47	4.48	10.09	11.12	6.06

Table S4: Multi-model mean changes and inter-model uncertainty (standard deviation) in streamflow under SSP1-2.6, SSP5-8.5 for the Near (2021-2040), Mid (2041-2060), and Far (2081-2100) terms, Related to STAR Methods

Streamflow change (%)												
**Dam**	**Near period**				**Mid period**				**Far period**			
	**SSP1-2.6**		**SSP5-8.5**		**SSP1-2.6**		**SSP5-8.5**		**SSP1-2.6**		**SSP5-8.5**	
	**Mean**	**Std**	**Mean**	**Std**	**Mean**	**Std**	**Mean**	**Std**	**Mean**	**Std**	**Mean**	**Std**
TEHRI	13.95	27.94	5.96	22.96	10.73	34.27	19.80	25.50	13.06	33.72	28.96	24.82
RAMGANGA	10.18	24.81	-4.05	25.59	3.65	38.58	11.53	27.76	7.17	37.82	26.15	27.97
LAKHWAR	14.97	27.09	6.25	22.90	9.61	34.61	20.76	24.94	10.45	34.62	25.86	25.89
VYASI	17.16	28.29	9.25	25.38	11.14	35.00	23.77	27.40	12.05	36.21	28.74	26.90
KOTESHWAR	14.90	23.19	7.01	18.19	10.27	28.62	19.74	20.37	13.07	30.20	28.60	15.99
SRINAGAR	11.31	24.38	2.33	21.24	8.06	31.18	11.59	23.56	12.98	30.67	26.86	22.95
MANERIBHALI2	14.65	29.48	7.96	24.25	12.49	34.46	21.82	26.75	14.97	33.97	32.88	25.31
BHAKRA	11.83	22.18	7.45	15.74	10.95	19.25	16.47	18.85	11.62	21.31	29.37	15.17
PONG	13.45	18.89	10.69	16.69	10.98	17.53	16.56	17.92	10.31	21.06	23.03	15.64
THEIN	12.54	21.20	9.42	17.67	10.44	17.93	15.84	18.18	10.06	21.15	22.50	16.97
KOL	11.44	23.40	6.94	16.10	11.07	19.82	16.36	19.17	12.14	21.87	30.55	15.33
SALAL	9.28	23.60	3.42	16.09	7.31	18.23	6.16	17.51	8.17	21.14	11.46	15.26
CHAMERA1	12.48	21.39	9.28	17.72	10.42	17.67	15.72	18.21	10.15	21.20	22.39	16.41
CHAMERA2	12.75	20.68	9.84	17.55	10.86	17.48	16.44	18.01	10.65	20.87	23.34	15.73
CHAMERA3	12.68	20.58	9.70	17.45	10.83	17.37	16.15	17.91	10.75	20.76	23.07	15.38
RAMPUR	11.36	24.10	6.78	16.22	11.30	20.39	16.69	19.38	12.47	22.22	31.84	15.60
PARBATI2	10.96	20.52	7.25	16.92	9.03	16.45	10.41	18.48	10.87	20.57	16.85	14.62
NAPTHA	11.32	24.34	6.67	16.16	11.41	20.66	16.98	19.38	12.53	22.33	32.71	15.63
KARCHAM	11.29	24.34	6.61	16.05	11.44	20.73	17.12	19.30	12.50	22.31	33.09	15.58
RENGALI	4.15	22.49	-8.90	14.19	17.11	21.35	0.67	16.62	5.51	15.98	27.60	25.33
HIRAKUD	12.70	24.74	-6.94	22.21	23.19	23.84	1.76	21.32	16.46	22.89	35.79	25.73
BANGO	15.01	28.92	-9.53	24.42	19.29	30.40	5.97	29.21	22.21	30.67	41.60	32.02
KADANA	55.69	33.05	34.80	28.41	64.50	37.04	44.33	50.94	56.04	44.05	125.64	51.31
BARGI	17.61	25.80	-4.71	27.22	24.21	30.28	12.44	32.01	21.36	32.01	49.57	29.99
INDIRASAGAR	21.69	21.03	1.77	24.07	30.42	24.01	13.09	33.57	27.53	29.16	68.89	31.53
SARDARSAROVAR	25.33	18.23	5.67	22.90	35.89	20.20	15.51	28.78	31.44	26.31	81.96	31.85
OMKARESHWAR	22.70	17.73	3.30	22.56	31.51	19.56	13.55	29.09	28.95	24.92	70.91	29.90
MAHESHWAR	23.34	17.93	3.75	23.28	32.64	19.66	13.68	28.93	29.54	25.16	74.58	30.98
PANCHET	2.65	20.24	-0.29	15.25	15.20	20.57	10.65	16.82	0.81	18.49	39.24	27.68
MAITHON	4.71	22.26	5.16	16.05	17.76	24.91	17.94	18.87	1.14	21.21	51.02	28.97
UKAI	33.55	23.76	13.52	30.48	50.19	26.66	20.82	32.32	43.45	35.19	135.05	41.00
RIHAND	18.08	22.83	-6.82	22.76	19.79	30.70	8.35	25.04	21.01	27.41	46.64	32.77
MATATILA	23.29	31.83	11.03	28.27	27.60	28.11	20.10	52.77	32.10	38.11	71.77	37.11
RANAPRATAP	46.80	30.00	20.72	26.17	47.13	28.85	32.38	50.52	49.54	43.94	114.18	48.92
SRIRAMSAGAR	36.53	25.78	17.14	22.06	43.69	23.54	14.78	28.59	57.32	31.86	97.64	35.28
METTUR	9.40	28.01	10.75	31.71	15.17	29.68	4.41	30.40	11.77	27.37	29.24	35.34
NAGARJUN	17.12	22.63	5.37	15.87	28.73	24.80	6.16	24.66	16.18	25.91	50.88	25.60
SRISAILAM	16.96	24.10	5.64	16.57	28.59	26.93	6.28	25.98	16.45	27.63	50.51	26.36
ALMATTI	14.47	26.47	7.82	17.40	26.80	27.18	3.60	23.78	16.95	27.85	40.57	23.02
TUNGABHADRA	4.99	17.13	-0.63	17.52	17.17	26.47	-4.42	22.98	4.51	23.13	14.50	26.49
SHOLAYAR	2.13	19.04	1.84	18.26	5.91	15.35	-0.95	18.84	3.31	16.92	18.09	22.32
LINGANAMAKKI	1.38	15.64	-2.98	15.52	11.08	18.57	-7.19	16.38	2.08	16.62	-1.35	16.51
SUPA	3.03	17.28	-0.70	16.08	13.49	19.85	-4.69	16.39	4.56	19.06	5.51	18.26
GERUSOPA	0.87	8.23	-3.40	8.68	10.41	13.81	-7.71	8.85	1.61	13.32	-1.80	10.33
KODASALLI	3.09	13.07	-0.87	11.97	13.67	17.13	-5.38	12.64	4.05	16.49	6.39	14.37
KADRA	2.99	13.72	-1.08	12.53	13.37	17.57	-5.53	13.44	3.92	16.51	5.56	14.68

Table S5: Multi-model mean changes and inter-model uncertainty (standard deviation) in hydropower under SSP1-2.6, SSP5-8.5 for the Near (2021-2040), Mid (2041-2060), and Far (2081-2100) terms, Related to STAR Methods

Hydropower change (%)												
**Dam**	**Near period**				**Mid period**				**Far period**			
	**SSP1-2.6**		**SSP5-8.5**		**SSP1-2.6**		**SSP5-8.5**		**SSP1-2.6**		**SSP5-8.5**	
	**Mean**	**Std**	**Mean**	**Std**	**Mean**	**Std**	**Mean**	**Std**	**Mean**	**Std**	**Mean**	**Std**
TEHRI	33.90	14.95	25.55	12.09	24.13	22.52	34.57	10.59	24.30	16.07	58.71	5.33
RAMGANGA	30.35	39.25	2.54	29.23	7.02	47.33	31.94	35.29	19.48	43.69	58.87	23.07
LAKHWAR	26.90	28.18	17.79	20.90	14.29	31.73	39.61	24.23	14.50	34.47	45.23	19.09
VYASI	9.21	13.59	11.53	12.95	5.16	12.92	6.29	15.64	6.28	14.16	13.11	7.41
KOTESHWAR	15.80	14.53	12.89	13.39	9.81	17.67	20.66	12.96	12.57	17.37	29.25	11.12
SRINAGAR	5.99	15.78	7.04	15.22	1.55	14.58	4.15	16.44	6.56	16.50	11.89	13.02
MANERIBHALI2	6.89	13.93	10.03	13.24	4.08	11.97	5.48	13.42	7.21	13.22	11.83	9.34
BHAKRA	8.32	7.77	6.97	6.88	9.84	6.76	6.05	7.67	8.12	7.41	13.94	4.94
PONG	17.02	8.76	17.33	4.28	12.90	9.88	13.13	8.49	12.42	9.85	24.35	3.38
THEIN	13.97	14.74	11.02	14.67	10.67	13.54	10.99	14.21	10.05	16.92	21.34	12.46
KOL	7.13	19.13	6.41	14.62	8.49	14.20	6.05	14.22	8.84	14.34	17.88	11.45
SALAL	10.26	24.97	7.55	22.04	11.75	22.71	7.39	20.61	10.78	22.30	14.61	20.46
CHAMERA1	15.83	22.01	14.72	22.23	12.19	20.45	20.02	22.55	13.05	25.05	27.23	19.08
CHAMERA2	3.16	10.79	0.52	9.87	3.82	10.03	-0.34	7.42	3.67	8.81	3.06	7.91
CHAMERA3	3.37	11.33	0.77	10.29	4.06	10.21	-0.36	7.62	3.85	9.31	3.41	8.38
RAMPUR	2.58	5.72	1.09	5.29	2.84	5.44	2.44	4.94	2.98	4.88	4.82	4.38
PARBATI2	11.10	17.31	8.84	16.23	10.36	15.50	11.70	17.42	11.07	18.09	17.92	13.48
NAPTHA	2.76	6.18	1.13	5.51	2.98	5.93	2.62	5.33	3.15	5.28	5.37	4.60
KARCHAM	2.75	6.11	1.13	5.40	2.96	5.83	2.62	5.23	3.16	5.19	5.36	4.51
RENGALI	8.44	12.31	-1.61	11.56	13.27	12.84	5.71	16.60	11.14	11.21	16.25	15.96
HIRAKUD	6.35	3.31	2.05	7.15	5.68	4.12	4.33	5.71	6.97	4.95	8.95	5.03
BANGO	23.53	32.48	-2.19	35.68	31.06	25.95	22.51	28.08	31.77	25.48	45.95	20.68
KADANA	38.96	8.47	24.62	12.49	39.54	15.80	17.37	21.37	33.24	14.76	62.77	10.97
BARGI	14.57	18.56	4.98	17.39	14.37	12.43	13.95	19.54	14.65	16.36	32.03	11.74
INDIRASAGAR	16.30	18.27	-3.00	19.57	18.20	10.78	6.91	25.70	18.83	19.64	49.72	13.96
SARDARSAROVAR	11.16	8.87	0.92	9.69	12.85	6.83	5.32	14.09	12.92	10.52	31.21	6.99
OMKARESHWAR	17.77	17.83	-2.62	19.50	20.85	12.12	9.14	25.50	20.98	19.83	55.77	15.45
MAHESHWAR	19.69	18.47	-2.69	20.90	23.61	13.42	9.96	27.50	23.15	21.83	61.64	16.16
PANCHET	5.88	8.51	4.02	11.32	8.23	9.90	9.36	9.70	3.76	12.59	13.52	16.91
MAITHON	7.62	10.09	6.36	11.49	8.34	10.68	12.16	10.29	2.88	14.95	16.39	18.85
UKAI	22.16	9.04	1.90	9.81	34.11	5.11	10.59	11.52	15.51	19.68	47.89	5.06
RIHAND	32.85	12.48	-7.80	30.32	37.08	9.95	20.38	12.64	35.93	7.85	48.11	8.02
MATATILA	-2.16	11.27	-3.82	9.29	-5.24	10.60	-3.68	14.19	-2.46	10.68	2.82	5.44
RANAPRATAP	18.62	13.10	11.30	12.24	18.21	11.24	1.82	17.82	12.81	20.63	31.00	4.08
SRIRAMSAGAR	29.97	13.96	12.86	8.41	38.30	10.41	17.97	16.49	29.34	28.25	60.90	10.77
METTUR	4.84	15.20	-0.84	12.19	6.93	14.40	-8.42	18.64	3.00	15.22	8.23	19.72
NAGARJUN	22.05	12.56	8.88	14.89	29.95	8.22	1.95	16.39	13.63	28.61	41.26	4.70
SRISAILAM	17.74	9.28	7.37	10.36	24.16	7.65	1.47	12.22	11.66	22.56	35.83	5.87
ALMATTI	17.35	13.99	8.78	11.94	22.74	16.02	3.28	11.47	11.90	24.01	39.04	9.43
TUNGABHADRA	9.54	11.07	0.87	10.81	12.69	13.52	-9.12	15.58	5.52	19.28	10.86	14.96
SHOLAYAR	3.36	23.21	-0.57	20.16	9.12	19.73	-4.66	20.78	5.18	20.50	29.36	18.88
LINGANAMAKKI	4.79	11.54	-3.10	12.03	11.23	10.29	-12.5	16.29	0.52	17.08	-3.25	12.86
SUPA	10.09	25.20	-6.22	28.98	58.20	32.85	-23.9	35.22	17.62	39.92	21.30	24.65
GERUSOPA	1.53	14.05	-5.70	15.53	14.77	16.42	-13.6	17.45	1.16	20.04	-5.05	17.36
KODASALLI	6.18	21.66	-2.05	20.15	17.78	21.25	-9.18	20.87	5.96	23.44	8.71	20.74
KADRA	5.50	23.09	-2.34	21.10	18.68	23.16	-8.78	22.08	6.44	23.55	6.67	22.18

Table S6: Overview of the warming levels and in which year of the decade they are reached in the corresponding GCM, Related to STAR Methods

**Overview of the warming levels and in which year of the decade they are reached in the corresponding GCM**						
CMIP6 Models	SSP	1º	1.5º	2º	2.5º	3º
BCC-CSM2-MR	SSP1-2.6	2028	NaN	NaN	NaN	NaN
	SSP5-8.5	2023	2036	2048	2058	2069
INM-CM5-0	SSP1-2.6	2031	NaN	NaN	NaN	NaN
	SSP5-8.5	2028	2042	2056	2069	2085
MIROC6	SSP1-2.6	2041	NaN	NaN	NaN	NaN
	SSP5-8.5	2032	2045	2060	2069	2079
NorESM2-MM	SSP1-2.6	2035	NaN	NaN	NaN	NaN
	SSP5-8.5	2029	2039	2056	2067	2076
TaiESM1	SSP1-2.6	2017	2025	2039	2076	NaN
	SSP5-8.5	2018	2026	2033	2041	2050

Table S7: Details of the General Circulation Models (GCMs) used in the study, Related to STAR Methods

**S. No.**	**Model name**	**Description**	**Reference**	**Spatial resolution (km) [lon × lat]**	**Components**
1	BCC-CSM2-MR	Beijing Climate Center, Beijing (China)	Wu et al. (2018)	320 × 160	atmosphere, land, ocean, sea ice
3	INM-CM5-0	Institute for Numerical Mathematics, Russian Academy of Science, Moscow (Russia)	Volodin et al. (2019)	180 × 120	aerosol, atmosphere, land, ocean, sea ice
4	MIROC6	Japan Agency for Marine-Earth Science and Technology (Japan)	Tatebe et al. (2019)	256 × 128	aerosol, atmosphere, land, ocean, sea ice
5	NorESM2-MM	Climate Modeling Consortium consisting of Center for International Climate and Environmental Research (Norway)	Seland et al. (2019)	288 × 192	aerosol, atmosphere, atmosphere Chem, land, land ice, ocean, ocean Bgchem, sea ice
6	TaiESM1	Research Center for Environmental Changes, Academia Sinica (Taiwan)	Lee et al. (2020)	288 × 192	aerosol, atmosphere, atmosChem, land, ocean, sea ice

Table S8: Bias (GCM-OBS) in average precipitation and temperature over India, Related to STAR Methods

GCM	Bias (precipitation, %)	Bias (temperature, ºC)
BCC-CSM2-MR	0.60	-1.41
INM-CM5-0	7.64	-3.53
MIROC6	2.21	1.55
NorESM2-MM	-8.40	-0.97
TaiESM1	8.94	-1.36
**After bias-correction**		
**GCM**	**Bias (precipitation, %)**	**Bias (temperature, ºC)**
BCC-CSM2-MR	1.83	-0.065
INM-CM5-0	-3.39	-0.051
MIROC6	2.61	-0.122
NorESM2-MM	3.55	-0.095
TaiESM1	2.11	-0.019

Table S9: Coefficient of determination (R^2) and Nash Sutcliffe Efficiency (NSE) values at selected streamflow gauging station for 13 river basins during the calibration and validation periods, Related to STAR Methods

Basin	Gauging Station	Calibration			Validation		
		Period	R^2	NSE	Period	R^2	NSE
**South Coast**	Neeleshwar	1991-2000	0.73	0.69	2006-2014	0.61	0.59
**Narmada**	Handia	1991-2000	0.76	0.73	2007-2016	0.74	0.67
**Tapi**	Sarangkheda	1991-1998	0.65	0.64	1999-2006	0.76	0.67
**Mahi**	Chakaliya	1992-2000	0.6	0.55	2006-2010	0.7	0.61
**West Coast**	Bantwal	1991-1999	0.88	0.77	2007-2016	0.77	0.69
**Subarnarekha**	Govindpur	1994-2003	0.63	0.62	2004-2013	0.71	0.71
**Brahmani**	Jenapur	1991-2000	0.65	0.58	2001-2010	0.67	0.63
**Ganga**	Farakka	1951-1960	0.79	0.79	1965-1973	0.84	0.84
**Mahanadi**	Basantpur	1991-2000	0.75	0.71	2005-2014	0.7	0.65
**Godavari**	Polavaram	1981-1990	0.82	0.80	1991-2000	0.78	0.73
**Cauvery**	Kollegal	1997-2002	0.54	0.52	2006-2010	0.58	0.58
**Krishna**	Harallihalla	1991-2000	0.78	0.77	2001-2010	0.74	0.66
**Indus**	Mukesar	1968-1972	0.67	0.61	1973-1979	0.69	0.65
